# Accurate geometrical restraints for Watson–Crick base pairs

**DOI:** 10.1107/S2052520619002002

**Published:** 2019-03-27

**Authors:** Miroslaw Gilski, Jianbo Zhao, Marcin Kowiel, Dariusz Brzezinski, Douglas H. Turner, Mariusz Jaskolski

**Affiliations:** a Department of Crystallography, Faculty of Chemistry, A. Mickiewicz University, Poznan, 61-614, Poland; b Center for Biocrystallographic Research, Institute of Bioorganic Chemistry, Polish Academy of Sciences, Poznan, 61-704, Poland; c Department of Chemistry, University of Rochester, Rochester, NY 14627, USA; d Center for RNA Biology, University of Rochester, Rochester, NY 14627, USA; e Institute of Computing Science, Poznan University of Technology, Poznan, 60-965, Poland; f Center for Artificial Intelligence and Machine Learning, Poznan University of Technology, 60-965, Poland

**Keywords:** stereochemical restraints, nucleobase geometry, Protein Data Bank (PDB), Cambridge Structural Database (CSD), quantum-mechanical calculations, ultrahigh resolution, canonical Watson–Crick base pairs, isocytosine (iC), isoguanine (iG)

## Abstract

Geometrical restraints provide key structural information for the determination of biomolecular structures at lower resolution by experimental methods such as crystallography or cryo-electron microscopy. In this work, restraint targets for nucleic acids bases are derived from three different sources and compared: small-molecule crystal structures in the Cambridge Structural Database (CSD), ultrahigh-resolution structures in the Protein Data Bank (PDB) and quantum-mechanical (QM) calculations. The best parameters are those based on CSD structures. After over two decades, the standard library of Parkinson *et al.* [(1996), *Acta Cryst*. D**52**, 57–64] is still valid, but improvements are possible with the use of the current CSD database. The CSD-derived geometry is fully compatible with Watson–Crick base pairs, as comparisons with QM results for isolated and paired bases clearly show that the CSD targets closely correspond to proper base pairing. While the QM results are capable of distinguishing between single and paired bases, their level of accuracy is, on average, nearly two times lower than for the CSD-derived targets when gauged by root-mean-square deviations from ultrahigh-resolution structures in the PDB. Nevertheless, the accuracy of QM results appears sufficient to provide stereochemical targets for synthetic base pairs where no reliable experimental structural information is available. To enable future tests for this approach, QM calculations are provided for isocytosine, isoguanine and the iCiG base pair.

## Abstract 2

Revised geometrical parameters are proposed for the Watson–Crick pairs of nucleobases, for use as restraints in modeling and refinement of the structures of nucleic acids. Accurate values of these parameters were derived (and compared) from small-molecule Cambridge Structural Database structures, from super accurate ultrahigh-resolution nucleic acid structures in the Protein Data Bank, and from quantum mechanical calculations. The effect of base pairing on the molecular geometry of the nucleobases is also investigated.

## 1. Introduction   

### 1.1. Motivation   

**Figure 1:**
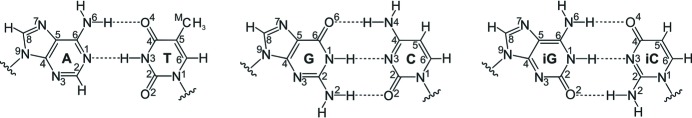
Covalent structure of the WC base pairs discussed in this work, with standard atom numbering. Hydrogen atoms are marked to define the covalent/tautomeric topology but they are not numbered as they are not included in the generated libraries of stereochemical restraints. The AU base pair (not shown) has the same atom numbering as the AT pair, except that it does not have the CM methyl substituent at the pyrimidine (U) base. Hydrogen bonds are marked by dashed lines.

Methodological advances in experimental methods for biomolecular structure determination coupled with rapid increase of the volume of the information stored in the Protein Data Bank (PDB) (Berman *et al.*, 2000 ▸) and Cambridge Structural Database (CSD) (Groom *et al.*, 2016 ▸) provide manifold motivations for this paper, which is the second part of our series reinvestigating stereochemical restraints for nucleic acids structure (Kowiel *et al.*, 2016 ▸). (i) Firstly, we were interested in checking whether the standard nucleobase restraints derived by Parkinson *et al.* (1996 ▸) from the small-molecule data in the CSD might need revision, after more than two decades and with a nearly tenfold expansion of this database. (ii) Secondly, we wanted to investigate whether the accuracy of nucleobase geometry derived from modern quantum mechanical (QM) calculations is comparable to or perhaps even better than the quality of the experimental geometry derived from crystallography. (iii) Thirdly, we were interested in checking whether the restraints derived from unpaired bases are sufficiently adequate to describe the molecular geometries of base pairs. Intuitively, there are reasons to believe that there should be geometrical differences between paired and isolated nucleobases, as the inter-base hydrogen bonds would certainly influence (even if only to a small degree) the electronic structure of the aromatic systems. Such consequences of base pairing are not a new concept. They have been analyzed, for example, from the point of view of the aromaticity of isolated and paired bases (Cyrański *et al.*, 2003 ▸). (iv) Fourthly, the above considerations are important for approximations inherent in molecular dynamics (MD) simulations because parameters for the bases are derived from QM calculations (Smith *et al.*, 2017 ▸; Šponer *et al.*, 2018 ▸). (v) Finally, we had a practical problem of missing reliable geometrical restraints for non-canonical bases, such as isocytosine and isoguanine, which are present in some crystal structures we are studying to enhance the information available so far only from NMR spectroscopy (Chen *et al.*, 2007 ▸). Isocytosine (iC) and isoguanine (iG) are analogous to their parent bases (C and G, respectively), but have the key amino and keto substituents swapped at the aromatic systems (Fig. 1 ▸). This leads to the possibility of iCiG base pair formation with three hydrogen bonds as in the Watson–Crick (WC) CG base pair but with the polarity of these interactions inverted. Obviously, the electronic structure of the iCiG and CG base pairs is quite different, leading to different molecular dimensions of these systems.

Accurate stereochemical information on biological macromolecules in the form of geometrical restraints (when applied softly), or sometimes constraints (when applied as fixed geometry), is a necessary ingredient of macromolecular structure determination. Stereochemical restraints are usually applied at the stage of model refinement or optimization by such experimental methods as cryo-electron microscopy, NMR spectroscopy, and most notably X-ray crystallography. Such restraints are crucial when the volume of experimental observations, especially at low resolution, is insufficient to define the macromolecular geometry by reference to experimental data alone. Historically, different compilations of stereochemical restraints, defined as restraint targets (*i.e.* values) and their standard deviations (*i.e.* error estimates), have been presented, but the most prevalent approach is to derive such restraints from the analysis of accurately determined small-molecule crystal structures collected in the CSD. For nucleic acids, the currently used restraint dictionary was compiled by Parkinson *et al.* (1996 ▸) more than 20 years ago.

In this work, the discussion of molecular geometry is restricted to bond distances (*d*) and angles (*a*). We consider the aromatic nucleobases to be essentially flat and recommend adequate planarity restraints as currently implemented in popular refinement programs.

### 1.2. The different sets of restraint targets tested in this work   

The analyses presented in this work are based on comparisons of the following sets of restraint targets (*i.e.* of molecular geometry) of nucleobases:

I: CSD-based

Ia: The classic targets presented by Parkinson *et al.* (1996 ▸).

Ib: Molecular geometry derived from the current version of the CSD.

II: PDB-based

Two ultrahigh-resolution crystal structures, refined without the influence of stereochemical restraints: 1d8g (0.74 Å) B-DNA comprised of C, G, A, T bases (Kielkopf *et al.*, 2000 ▸); and 3p4j (0.55 Å) Z-DNA comprised of C and G bases (Brzezinski *et al.*, 2011 ▸).

III: QM-based

The subdivision in this group is dual. Firstly, results from calculations by three different variants of QM methods are considered: M06-2X, B3LYP-D3, and B3LYP-D3(BJ) (see below). The results of the three methods are compared among themselves (Table S1), but for comparisons with other groups of restraints (I, II) only the BJ results are used because they are considered to be the best in the QM group (III). Secondly, the QM calculations are presented for:

IIIa: Isolated nucleobases (A, C, G, T, U, iC, iG);

IIIb: Nucleobases included in the WC base-pairing context. A special case is presented by adenine (A), whose geometry is derived in two ways: from the AT and AU base pairs.

Note that for the purpose of consistent covalent structure the QM models were N-substituted by a methyl group mimicking the glycoside linkage (connection to the ribose ring). In the actual geometry analyses, however, the glycosidic bond geometry was not included, as it will be treated together with the sugar moiety restraints in the next paper of this series.

## 2. Materials and methods   

### 2.1. The analytical tools employed for data comparisons   

Direct comparisons of different sets of geometrical parameters were carried out with application of the concept of root-mean-square deviation (RMSD), which is the reference parameter used for evaluation of the agreement of PDB models with standard (‘ideal’) geometry. RMSD values can be calculated for a wide range of geometrical parameters. However, in the present analyses, the RMSD method was applied (separately) only to bond lengths (*d*) and to bond angles (*a*). For instance, if the bond lengths in guanine (*G*) residues from an ultrahigh-resolution PDB structure *S* (II) were to be compared with the target values in the Parkinson library (Ia), we would list side-by-side all the corresponding C—C, C—N, and C—O bond lengths in the two models denoted as 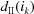 and 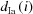, where *i* ∈ Bonds (*G*) is the set of analyzed bonds (12 in the case of guanine) and 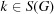 is the set of guanine instances in the PDB structure *S* (*e.g.* six for 3p4j). Then we would calculate the RMSD parameter for bond distances as: 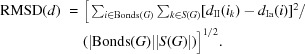
Analogously, the RMSD for bond angles would be computed as: 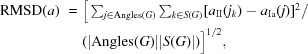
where |*X*| is the number of elements in a given set *X*. The RMSD criterion compares, therefore, two sets of numerical values. Typically, one of the sets contains some stereochemical targets against which a set of observed values is to be assessed. In this study, we will use RMSD mainly to compare mean CSD-based geometries (I) or QM-based parameters (III) against sets of bond distances and angles observed in ultrahigh-resolution PDB structures (II) refined without the influence of stereochemical restraints.

If the compared values in at least one set come from experiment, the significance of the RMSD criterion can be assessed with reference to the intrinsic uncertainties of the compared values. For example, if the bond distances come from an accurate crystallographic experiment and have intrinsic uncertainties of ∼0.005–0.010 Å, then an RMSD(*d*) value of 0.03 Å would be considered significant (as exceeding the uncertainty at least three times), while a value of 0.003 Å would not. In some situations such a reference to an ‘internal standard’ is not possible (*e.g.* when comparing two sets of theoretical results) and then we usually assess the significance of the RMSD with reference to the level of error in a comparable experiment. Please note that the RMSD criterion is a global indicator, whose large value can signal a problem without pinpointing its source.

Despite its apparent simplicity, the present analysis is in fact quite complex. Not only do we have different sets of parameters (bonds, angles) to compare, but also results from different methods (I, II, III) and their subvariants. In addition, the comparisons have to be carried out separately for each of the nucleobases (A, C, G, T, U, iC, iG), but finally, if possible, some more general colligations would be expected. The RMSD method should provide a useful tool for generalizations, so that this multidimensional comparison exercise does not get out of hand.

In keeping with the convention used in crystallography, when appropriate standard deviations for statistically distributed values are available, they are given in parentheses, following the mean value, with units of the last significant digit of the mean.

### 2.2. Selection of CSD fragments   

Sets of high-resolution structures containing the five nitro­genous bases: cytosine (C), thymine (T), uracil (U), adenine (A), and guanine (G) (Fig. 1 ▸), were collected from the CSD version 5.39 update 3 using *CONQUEST 1.33* (Bruno *et al.*, 2002 ▸). The CSD Python API 1.5.3 (Groom *et al.*, 2016 ▸) was used to compute geometrical parameters, which were later averaged to yield the desired restraint targets and their standard deviations, as listed in the CSD columns of Tables 1 ▸, 2 ▸, 3 ▸, 4 ▸ and 5 ▸. Structure selection criteria were established on the basis of both chemical and crystallographic considerations. In particular, protonated bases were rejected by considering only covalent topology consistent with the canonical tautomers of the nucleobase molecules, as presented in Fig. 1 ▸. Only pyrimidines substituted with carbon atom at N1 and purines substituted at N9 were selected. Crystal structures of transition metal complexes were explicitly excluded from the queries.

Only structures with *R* ≤ 6% and average estimated standard deviation (e.s.d.) of C—C bond lengths [σ(C—C)] < 0.01 Å were selected, based on the statistical analysis presented in the next section. These selection criteria are similar to those used earlier by Parkinson *et al.* (1996 ▸). To minimize the standard deviations in the target bond distances and angles, a modified Z-score test (Kowiel *et al.*, 2016 ▸; Iglewicz & Hoalgin, 1993 ▸) was used to identify and reject outliers. In this test, a data item 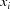 (in our case a bond distance or angle) is treated as an outlier if 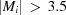. 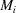 is calculated as follows:
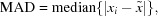


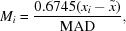
where 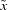 denotes the median of the sample. In the analyses described in the subsequent sections, when a parameter (bond distance or angle) in a given CSD structure was earmarked as an outlier, the entire CSD entry was removed from all calculations as potentially contaminated by gross error. In the final database of examples, there were 147 C bases, 364 T bases, 180 U bases, 216 A bases, and 63 G bases. For comparison, the library of Parkinson *et al.* (1996 ▸) was compiled using 28 C bases, 50 T bases, 46 U bases, 48 A bases and 21 G bases. The CSD codes of structures selected for this study are listed in supplementary Table S2.

### 2.3. CSD sampling methodology   

The structure sampling criteria presented in the previous section were established based on statistical analyses of the distributions of bond lengths and angles retrieved using varying quality restrictions. To guide the analysis, we focused on two statistics describing the samples: (i) the standard error of the mean (SEM), which assesses the confidence of the estimated mean value of a given geometrical parameter (bond length/angle); and (ii) the sample standard deviation, which describes the scatter in the sample. Our goal was to find such selection criteria which produce the smallest SEM and standard deviation. To learn which quality metrics are crucial for achieving this goal, we analyzed CSD samples with varying (i) maximum *R*-factor, (ii) maximum σ(C—C), (iii) all structures/only non-disordered structures, and (iv) all structures/structures after outlier removal.

**Figure 2:**
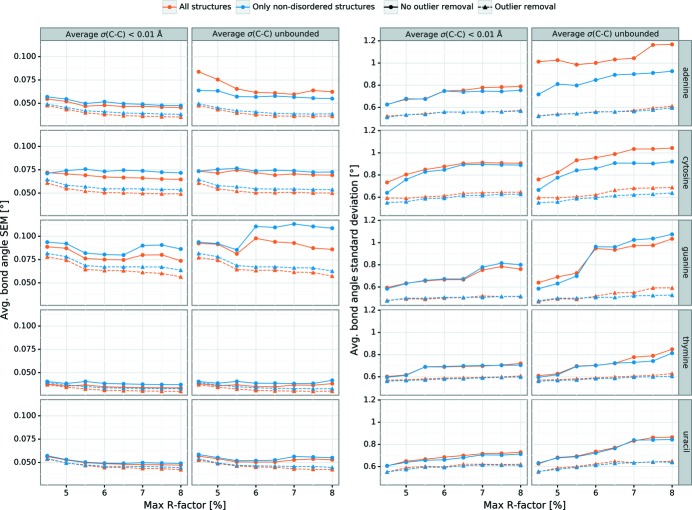
Analysis of average bond angle (°) standard error of the mean (SEM) (left) and sample standard deviation (right) for varying CSD sampling criteria: maximum *R*-factor (*x*-axis), maximum σ(C—C) (columns), all/non-disordered structures (blue/orange), and all structures/outlier removal (solid/dash line).

First, we analyze how the average SEM (Fig. 2 ▸, left) and standard deviation (Fig. 2 ▸, right) of bond angles change with maximum *R* (*x*-axis), increasing from 4.5% to 8.0% in steps of 0.5%. The general trends of the SEMs and standard deviations are related to the maximum *R*-factor threshold; for most bases (panel rows), the higher the maximum *R*-factor, the smaller the SEM and the wider the standard deviation, regardless of other selection criteria. One can also notice that by using only non-disordered structures, one achieves merely a slightly smaller standard deviation, but a worse approximation of the mean. Indeed, manual inspection of the CSD entries shows that disorder in the rejected structures is almost exclusively found outside of the queried substructure (base). Therefore, under this criterion, it is more profitable to also include disordered entries to obtain a larger sample (supplementary Fig. S1). Moreover, it seems that limiting the sample to structures with σ(C—C) below 0.01 Å (Fig. 2 ▸, panel columns) offers a slightly better approximation of the mean than ignoring this quality criterion. However, the most important gain, both in terms of SEM and standard deviation, is achieved by using the outlier removal method presented in the previous section (Fig. 2 ▸, dashed lines). If one were to use only one method for sample selection for CSD-based restraints, it should be the outlier removal procedure. Similar relations were observed for bond lengths (supplementary Fig. S2).

The maximum *R*-factor in our samples was selected as a compromise between the SEM and standard deviation. We chose *R* ≤ 6% as for this threshold the SEM seems to level out for most of the bases. Moreover, the mean values themselves are stable up to around *R* ≤ 6% and start to diverge with the inclusion of less accurate (higher *R*) structures (supplementary Fig. S3). Therefore, using a higher *R* value would result in a similar (albeit not identical) approximation of the mean with a higher standard deviation of this mean. This choice was further confirmed by the *F* test (with significance level α*_F_* = 0.05) used to compare the variance of the reference set with *R* ≤ 8%, with the variances of sets with lower *R*-factor thresholds. Although the results of this test are not uniform for all bases, *R* ≤ 6% is the value for which most of the bases have a significantly different variance (supplementary Fig. S4). We note that a similar conclusion was reached by Parkinson *et al.* (1996 ▸); yet our selection also involves the outlier removal procedure, which, as shown above, is crucial for obtaining a reliable sample.

We also verified the frequency distributions of bond lengths and angles for each queried base (supplementary Fig. S5–S9). Some of the bond length/angle distributions are significantly different from the normal distribution according to the Shapiro–Wilk test (Shapiro & Wilk, 1965 ▸) with significance level α*_S_* = 0.05; nevertheless, all the distributions are unimodal. The deviations from the normal distribution are mostly due to skewness (*e.g.* adenine C4—C5 bond; supplementary Fig. S5). However, our analysis revealed only 11 non-normal distributions, compared to 27 such cases noted by Parkinson *et al.* (1996 ▸). This shows the importance and power of the nearly fivefold larger sample size used in our study. Finally, the bond length/angle distributions for structures determined at higher (≥ 150 K) or lower (< 150 K) temperatures are similar (not shown). However, currently the majority of cases fall in the former category.

### 2.4. Quantum mechanical calculations   

Test calculations were performed on adenosine, guanosine, and their equivalents with a methyl group replacing the ribose. All structures were optimized without constraints. Both optimized nucleosides have C3′-*endo* sugar pucker, and base in *anti* orientation. Bond lengths and angles are shown in supplementary Table S3. RMSD values between adenosine and methyl­ated A for bond lengths and angles are 0.0045 Å and 0.213°, respectively. Equivalent RMSDs between guanosine and methyl­ated G are 0.0063 Å and 0.187°. Evidently, a methyl group is sufficient to mimic effects of a sugar on the covalent structure of a base.

Based on the above tests, all bases and base pairs in additional QM calculations had sugar mimicked with a methyl group. Each system was optimized with three methods in vacuum. Thus, the calculations concentrate on the fundamental interactions within bases and base pairs without being restricted to an environment specific to any individual crystal structure or sugar type, *etc*. To test that all optimized structures are the global minima, vibrational frequencies were calculated using the same method. All calculations were performed using the *Gaussian 09* package (Frisch *et al.*, 2013 ▸).

Although there is no phosphate–ribose backbone or base stacking interactions in these systems, there are hydrogen bonds in the base pairs; therefore, it is important that dispersion interactions are accurately calculated to get reliable geometries. Three methods were used in this work, which are known to yield reasonably accurate results for nucleic acids (Kruse *et al.*, 2015 ▸): B3LYP-D3 with atom pairwise D3 dispersion correction (Grimme *et al.*, 2010 ▸), B3LYP-D3(BJ) with Becke–Johnson damping function (Grimme *et al.*, 2011 ▸), and M06-2X, which includes medium-range dispersion interactions (Zhao & Truhlar, 2008 ▸). All calculations were performed at the triple zeta basis set aug-cc-pVTZ level of theory.

## 3. Results   

In the following subsections, we present a number of comparisons of results obtained using the different sources of structural information (I, II, III) as outlined above. We end each of the comparisons with a succinct conclusion. Those partial conclusions are recapitulated in the *Discussion* [Sec sec4], which provides a general summary.

### 3.1. Consistency of the results obtained by three different QM methods   

The QM-optimized geometries from three methods are similar and show only very small numerical differences (supplementary Table S1). For example, the RMSDs between BJ and M06 are only ∼0.005 Å/0.24° for bond distances/angles, respectively. However, the paired bases show slightly larger RMSD values for distance than isolated bases. This is expected because the BJ method is able to describe long-range dispersion interactions better than M06, and this has more pronounced effects on systems with hydrogen bonds, *i.e.* base pairs. On the other hand, adding the Becke–Johnson damping function does not change the geometries significantly. As shown in Table S1, the RMSDs between D3(BJ) and D3 without BJ are as small as 0.001 Å/0.09°. For both isolated and paired bases, the difference between D3(BJ) and D3 without BJ is negligible. Of the three methods we, therefore, focus on D3(BJ).

Conclusion: The three QM methods provide very similar optimized structures. For both isolated and paired bases, the differences between methods are not greater than experimental errors.

### 3.2. Comparison of QM results (BJ) for isolated and WC-paired bases   

The differences of QM-calculated geometry *between* isolated and paired bases are quite significant, with RMSD(*d*) of 0.010–0.018 Å and RMSD(*a*) of 0.94–1.29° (Tables 2 ▸, 3 ▸, 4 ▸, 5 ▸, 6 ▸ and 7 ▸), except for adenine, where they have no significance, regardless of the reference base pair, AU or AT (Table 1 ▸). In the latter case (adenine base pairs) the values of RMSD(*d*) ≤ 0.008 Å and RMSD(*a*) ≤ 0.6° are at or below the level of experimental errors for these parameters (for AT as low as 0.0006 Å/0.08°). The lack of pairing perturbation of A may reflect the facts that AU and AT pairs have only two hydrogen bonds whereas GC pairs have three. Moreover, A has a larger aromatic system than U or T to distribute geometrical perturbations.

For bases other than A (*i.e.* C, G, U, T, iC and iG), the situation is consistently different, as the respective RMSDs between QM-calculated values for isolated and paired bases are about two times higher. Actually, the highest differences are noted for the iso forms iC and iG (Tables 6 ▸ and 7 ▸).

Conclusion: On its face value, such a result would in general seem to reinforce the notion that the geometry of isolated and paired bases is sufficiently different (with the possible exception of adenine) to justify the derivation of restraint targets for nucleic acid duplexes from base pairs rather than from isolated bases.

### 3.3. On the use of high-resolution experimental PDB models for comparisons   

To assess the reliability of different compilations of stereochemical restraint targets, we use as reference the molecular dimensions of the highest-resolution nucleic acids structures in the PDB. In a sense, this approach is opposite to what is normally done during macromolecular structure refinement, where a (lower resolution) experimental model is gauged against ‘ideal’ stereochemical targets. The PDB version of January 20, 2019, contains only seven nucleic acids structures determined to at least 0.8 Å resolution, corresponding to typical level of resolution in small-molecule crystallography (5jzg, 4ocb, 4hig, 3p4j, 1j8g, 1i0t, 1d8g). The key reference model is the highest-resolution nucleic acid structure in the PDB (3p4j) determined at 0.55 Å for Z-DNA without any restraints whatsoever imposed on the nucleic acid geometry (Tables 2, 5). As noted by the original authors (Brzezinski *et al.*, 2011 ▸), the nucleotide molecular geometry of 3p4j is highly regular with very small deviations in the measurements of the same stereochemical parameters. For instance, the scatter of the nucleobase bond length/angle determinations is ∼0.003 Å/0.3°. Most of the other high-resolution PDB entries represent the same (4hig; *d*_min_ = 0.75 Å; *R* = 0.071; Drozdzal *et al.*, 2013 ▸) or very similar (4ocb; 0.75 Å; 0.122; Luo *et al.*, 2014 ▸) Z-DNA structures, sometimes with massive disorder (5jzg; 0.78 Å; 0.138; Drozdzal *et al.*, 2016 ▸) or poorly refined (1i0t; 0.60 Å; 0.160; Tereshko *et al.*, 2001 ▸), but always with explicit inclusion of geometrical restraints (not quite obvious for 1i0t). The disadvantage of the 3p4j structure as a reference is the absence of any nucleobases other than C or G. Therefore, we have also included as a reference the PDB structure 1d8g of B-DNA determined at 0.74 Å resolution (Kielkopf *et al.*, 2000 ▸), which is comprised of all the DNA bases, albeit with different frequencies (Tables 1 ▸, 2 ▸, 4 ▸ and 5 ▸). Despite a high degree of disorder of the sugar-phosphate backbone, the 1d8g model was also refined without explicit geometrical restraints and used only similarity restraints on the disordered moieties. Nevertheless, the scatter of the analogous molecular dimensions is much higher than for 3p4j, and for the C and G bases is calculated at 0.010–0.017 Å/0.52–1.13° (*i.e.* up to six/four times larger). This in itself illustrates the power of high resolution and the improvement of quality on extending the resolution from 0.74 to 0.55 Å, although other factors, such as disorder have to be taken into account as well (however, disorder would be expected to degrade resolution anyway). It is of note that one of the thymine bases modeled in the 1d8g coordinate set in dual conformation has been excluded from our analyses.

The seventh high-resolution PDB structure mentioned above (1j8g) corresponds to a tetraplex (*i.e.* not standard WC base-paired) RNA containing only U and G bases, refined at 0.61 Å with no mention of restraints (Deng *et al.*, 2001 ▸). That structure, however, despite superficial appearance of high quality, contains U36 with bogus atomic occupancy factors, ranging from 0.85 to 0.01, as well as other nucleotides with suspicious geometry (RMSD for nucleobase bonds versus Parkinson library of 0.048 Å). Therefore, that structure could not be used for validation.

Conclusion: The PDB contains only two ultrahigh-resolution structures suitable as sources of unbiased structural information for nucleic acids. It is always advantageous to use every bit of resolution to improve the quality (accuracy and precision) of crystallographic models.

### 3.4. Comparison of QM models with experimental nucleic acid geometry   

In the comparisons with 3p4j, the nucleobase QM models calculated for WC-paired CG bases are much closer to the experimental structure (where the bases are obviously WC-paired) than the QM models of isolated bases, as illustrated by the RMSD values which are roughly two times lower (Tables 2 ▸ and 5 ▸). Although the RMSD values characterizing the former case (bases in WC context) are quite respectable (∼0.011 Å/∼0.9°), they are inferior to those corresponding to experimental (CSD-derived) restraint dictionaries, as explained below.

Conclusion: QM calculations are able to correctly predict changes in nucleobase geometry arising from base pairing, in agreement with experimental observations for nucleic acids duplexes.

### 3.5. Comparison of experimental (CSD-derived) and QM molecular dimensions   

For the purpose of this comparison, the standard CSD-derived Parkinson library of restraint targets (Parkinson *et al.*, 1996 ▸) will be used. (It will be compared with the current CSD library in the next section.) Comparison of the Parkinson library with the experimental high-resolution PDB structures 1d8g and 3p4j (derived without its influence), and with the QM-derived parameters clearly demonstrates that the CSD-derived geometry adequately reflects the situation corresponding to paired rather than isolated nucleobases. This conclusion is based on the observation that the RMSD values calculated for the Parkinson library indicate a much better agreement with the QM calculations for base pairs (IIIb) than for isolated bases (IIIa) (Tables 1 ▸–5 ▸; Fig. S10).

At first, this conclusion seems puzzling, as one would expect the CSD geometry to be derived from organic moieties that, in general, are not involved in WC interactions. However, in the small-molecule crystal structures, from which the CSD geometry is derived, those moieties are certainly participating in abundant networks of intermolecular interactions that in all probability satisfy the hydrogen bonding potential of all the WC N and O centers of those moieties. Thus, even without formal involvement in WC pairing, the CSD moieties apparently mimic quite accurately the hydrogen bonding situation of such pairs. Moreover, the CSD data represent a variety of hydrogen-bonding situations (*e.g.* interactions with solvent molecules) rather than one rigid (even if optimized) theoretical model. Thus, the average CSD structure may reflect the actual DNA/RNA situation better as it provides the mean geometry of all possible configurations.

**Figure 3:**
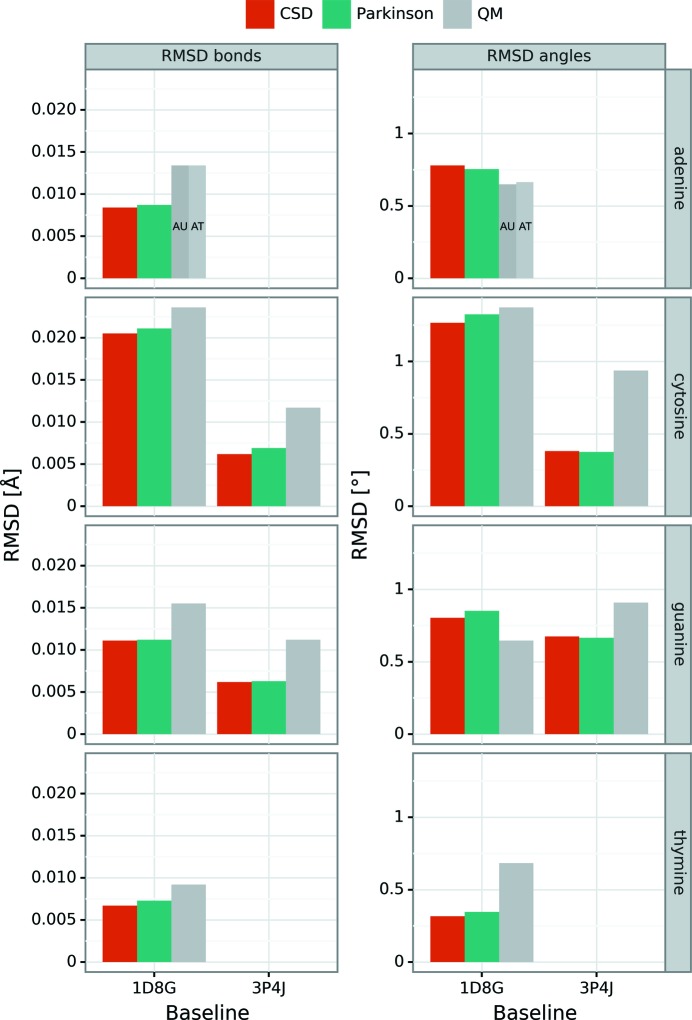
Comparison of RMSD values for bonds (Å, left) and for angles (°, right) calculated for the PDB structures 1d8g and 3p4j with reference to CSD-derived restraint targets compiled in the present work (red), restraints proposed by Parkinson *et al.* (1996 ▸) (green), and QM calculations (gray).

Another conclusion about the Parkinson parameters is that they are closer to the DNA reality than any parameters derived by QM calculations. The Parkinson parameters have RMSD values relative to 3p4j and 1d8g that are nearly half those for QM parameters (Fig. 3 ▸). The only exceptions are the angular parameters of the A and G bases of the 1d8g reference model, for which the QM RMSD values are lower (Tables 1 ▸ and 2 ▸, Fig. 3 ▸).

Conclusion: The CSD-derived restraint targets correctly reflect the WC base pairing context and are closer to reality than the restraint targets derived from QM calculations.

### 3.6. Validation of CSD-derived parameters   

A similar analysis as the one above but carried out for the parameters derived from the current version of the CSD database shows that the original Parkinson library is still remarkably valid and can be safely used. RMSD values for the current CSD set are marginally better than for the older set (Fig. 3 ▸) with very small variations of particular bonds/angles (Fig. S10). However, since in the Parkinson set there are a couple of numbers that deviate from the revised values at a level close to experimental errors (*e.g.* the C4—N4 bond length for cytosine or N2—C2—N1 angle for guanine), we recommend superseding the original library with the current version. For convenience, the revised version of the CSD-based library has been implemented in our RestraintLib server, as described below. Overall, it is remarkable how good the CSD-derived parameters are. With RMSD values of ∼0.006 Å/∼0.6° for 3p4j (or slightly more for 1d8g) they are much better than the level of model ‘ideality’ typically achieved in crystal structure refinements. The only exception is seen in the comparison with the cytosine geometry from 1d8g, where the RMSD values of ∼0.02 Å/1.27° are closer to typical macromolecular refinement results (Table 5 ▸). However, as mentioned above, this may reflect the level of accuracy of the reference model (1d8g) itself. The recommended values for use as CSD-based restraints (and as implemented in RestraintLib) are highlighted in bold in Tables 1 ▸–5 ▸. It is interesting to note that although our analysis included, on average, over five times more structures than used for the compilation of the Parkinson library, the standard deviations of the averaged geometrical parameters are generally the same. This suggests that these standard deviations reflect the intrinsic variability of the analyzed parameters, and not just the statistical precision of their estimation.

Conclusion: The original Parkinson library of nucleobase restraints is still generally valid although an improvement is possible by using the current version of the CSD. Our recommendation is to use the most up-to-date compilation of the restraints as presented in this paper and implemented in the RestraintLib server.

### 3.7. Recommended restraints for the iCiG base pair   

The standard library of restraints for nucleic acids structure compiled by Parkinson *et al.* (1996 ▸) does not contain unusual bases, such as isocytosine and isoguanine. On the other hand, the restraint dictionaries for such bases that are in circulation, *e.g.* as implemented in **REFMAC** (Murshudov *et al.*, 1997 ▸, 2011 ▸) (Tables 6 ▸ and 7 ▸), are rather suspicious and in our opinion should not be used for restraining crystallographic refinements. For example, all the valence angles generated by **REFMAC** for isocytosine are 120.0° (Table 6 ▸).

Given the increasing use of synthetic base pairs, it is likely that more of these will be included in macromolecular crystal structures. The results reported here suggest that QM calculations can provide sufficiently accurate stereochemical targets when none are available from experiments. The calculations on iCiG reported here provide a test for future applications of this approach. The iCiG pair has generated unexpected thermodynamics and structural effects, which are used to test computational predictions (Turner, 2013 ▸; Chen *et al.*, 2007 ▸). High resolution crystal structures would facilitate these tests by providing more detailed structural information, including sites of structured water.

Surprisingly, a search of the CSD reveals almost no structural information that could be used as a reliable source for generation of standard geometry for the iso forms of guanine and cytosine. For the isoguanine system, there are only chemically modified or/and protonated forms that are not suitable as iG templates. For iC, there is only one structure, ICYTIN01 (Portalone & Colapietro, 2007 ▸), that is of limited use, but even in this case the iC moiety is N1-protonated rather than N1-substituted and is involved in the formation of a hemiprotonated iCiC^+^ base pair, in which it is the (formally) neutral component. The iC moiety from the ICYTIN01 structure deviates (in terms of RMSD values for bonds/angles) from the **REFMAC** restraints by 0.037 Å/1.18°, from the single-base QM model by 0.028 Å/2.03°, and from the iCiG base pair QM model by 0.018 Å/1.21°, *i.e.* is in best agreement with the theoretical model derived from the iCiG base pair (Table 6 ▸).

As there is insufficient experimental information for statistically sound derivation of iso C and iso G geometry, we recommend using the QM parameters presented in Tables 6 ▸ and 7 ▸ as stereochemical restraints in refinement of iCiG base pairs. This recommendation is supported by the satisfactory agreement between QM calculations for Watson–Crick base pairs and the structures of 1d8g and 3p4j (Tables 1 ▸, 2 ▸, 4 ▸ and 5 ▸). Since the geometrical parameters obtained by QM calculations are not accompanied by estimates of uncertainty, we propose to use the average standard deviations characterizing the respective geometrical parameters derived experimentally (from CSD analysis) for the corresponding canonical bases (C/G): 0.009/0.008 Å for bond distances and 0.7/0.6° for bond angles.

Alternatively, there is a forthcoming ultrahigh-resolution crystal structure of double-stranded RNA with iCiG base pairs (M. Gilski *et al.*, in preparation) which could serve as a source of reliable restraint targets for refinement at lower resolution. The currently recommended restraints for the iso forms of cytosine and guanine are easily generated using the RestraintLib server and are highlighted in bold in Tables 6 ▸ and 7 ▸.

### 3.8. Availability   

The revised CSD-based restraints for nucleobase covalent geometry described in this paper (including the iso forms of cytosine and guanine) are highlighted in bold in Tables 1 ▸–7 ▸ and can be generated automatically using our RestraintLib server (http://achesym.ibch.poznan.pl/restraintlib/). The input is very simple and consists of a suitable PDB file containing nucleobases with standard labels (A, C, G, U, T, DA, DC, DG, DU, DT, IC, IG). The server will produce a file with all the bond length and bond angle restraints in **REFMAC** (Murshudov *et al.*, 1997 ▸, 2011 ▸), *PHENIX* (Adams *et al.*, 2010 ▸), or *SHELXL* (Sheldrick, 2015 ▸) format. Currently the RestraintLib server is capable of generating covalent restraints for the phospho­diester and nucleobase moieties. A future version will include the riboside moiety as well (work in progress).

## 4. Discussion   

Advanced QM calculations in *Gaussian 09* are capable of producing quite good molecular geometry for nucleobases, and the results are consistent across different parametrizations, provided a sufficiently high level of theory is used, such as the aug-cc-pVTZ basis set used in this study. In particular, the QM models correctly distinguish between isolated and WC-paired bases. The best source of reference geometry for paired bases are ultrahigh-resolution nucleic acid structures in the PDB. However, the nucleobase geometry derived from small-molecule crystal structures (of usually unpaired but hydrogen-bonded bases) in the CSD is also a realistic representation of the geometry found in WC pairs of nucleic acids duplexes. Thus, CSD-based compilations, such as the standard Parkinson library, or its updated version presented in this work and available for practical applications via our RestraintLib web server, are a legitimate source of restraint targets for macromolecular refinement. Moreover, on scrupulous pairwise comparisons with the reference PDB structures, the CSD parameters are still superior to those derived by QM calculations. However, for non-canonical bases, such as iC and iG, for which no reliable experimental structural information is available, the QM geometry is currently the best source of stereochemical restraint targets.

The RMSD values calculated in the above analyses (at ∼0.006 Å/∼0.6° for 3p4j) between experimental data and the best set of restraint targets (current CSD-based) are lower than typically seen in nucleic acid crystal structure refinements. Since the RMSD parameters for the improved restraint targets of the phospho­diester group as proposed in the first paper of this series (Kowiel *et al.*, 2016 ▸) are also low [(O—)P—O 0.007 Å/0.54°and (P—)O—C 0.006 Å/0.97°], the inevitable conclusion is that the main source of stereochemical imperfection in crystallographic structures of nucleic acids is the sugar moiety. This aspect will be treated in the forthcoming paper of this series.

## Supplementary Material

Supporting Information in PDF format. DOI: 10.1107/S2052520619002002/lo5047sup1.pdf

## Figures and Tables

**Table 1:** Molecular geometry (Å, °) of N9-substituted adenine *N* denotes the number of fragments used to determine the parameters. The recommended restraints are printed in bold.

	PDB 1d8g (*N* = 2)	Parkinson (*N* = 48)	CSD (*N* = 216)	QM for A	QM for AU	QM for AT
N1—C2	1.337 (7)	1.339 (9)	**1.339 (7)**	1.338	1.348	1.348
C2—N3	1.328 (2)	1.331 (9)	**1.330 (7)**	1.331	1.333	1.334
N3—C4	1.340 (1)	1.344 (6)	**1.346 (6)**	1.334	1.344	1.344
C4—C5	1.384 (5)	1.383 (7)	**1.382 (8)**	1.394	1.400	1.400
C5—C6	1.404 (5)	1.406 (9)	**1.406 (8)**	1.405	1.414	1.413
C6—N1	1.349 (1)	1.351 (7)	**1.353 (7)**	1.340	1.355	1.355
C5—N7	1.394 (7)	1.388 (6)	**1.388 (7)**	1.380	1.385	1.384
N7—C8	1.301 (7)	1.311 (7)	**1.311 (7)**	1.309	1.316	1.316
C8—N9	1.356 (12)	1.373 (8)	**1.370 (8)**	1.377	1.383	1.383
N9—C4	1.375 (2)	1.374 (6)	**1.374 (7)**	1.375	1.378	1.377
C6—N6	1.338 (5)	1.335 (8)	**1.334 (7)**	1.349	1.343	1.343
C6—N1—C2	119.5 (5)	118.6 (6)	**118.6 (6)**	118.83	119.94	120.03
N1—C2—N3	128.4 (2)	129.3 (5)	**129.4 (7)**	128.36	127.85	127.78
C2—N3—C4	111.3 (3)	110.6 (5)	**110.5 (6)**	111.59	111.58	111.49
N3—C4—C5	126.4 (2)	126.8 (7)	**126.9 (6)**	126.56	126.82	126.95
C4—C5—C6	117.3 (2)	117.0 (5)	**117.1 (5)**	116.11	116.55	116.54
C5—C6—N1	117.0 (3)	117.7 (5)	**117.5 (5)**	118.55	117.26	117.21
N3—C4—N9	128.14 (2)	127.4 (8)	**127.2 (7)**	128.18	127.89	127.79
C6—C5—N7	132.4 (7)	132.3 (7)	**132.2 (6)**	132.99	132.59	132.47
C5—C4—N9	105.4 (2)	105.8 (4)	**105.9 (4)**	105.26	105.29	105.26
C4—N9—C8	106.2 (1)	105.8 (4)	**105.7 (4)**	106.00	106.11	106.09
N9—C8—N7	114.3 (8)	113.8 (5)	**113.9 (5)**	113.70	113.54	113.57
C8—N7—C5	103.8 (13)	103.9 (5)	**103.8 (4)**	104.14	104.20	104.09
N7—C5—C4	110.3 (6)	110.7 (5)	**110.6 (5)**	110.91	110.87	110.99
N6—C6—N1	119.5 (1)	118.6 (6)	**118.6 (7)**	119.09	119.40	119.42
N6—C6—C5	123.5 (2)	123.7 (8)	**123.9 (7)**	122.36	123.34	123.37
RMSD versus 1d8g (*d*/*a*)		0.0087/0.745	0.0084/0.779	0.0114/0.839	0.0134/0.649	0.0134/0.664
RMSD versus QM for A (*d*/*a*)					0.0080/0.561	0.0078/0.604
RMSD versus QM for AU (*d*/*a*)						0.0006/0.079

**Table 2:** Molecular geometry (Å, °) of N9-substituted guanine *N* denotes the number of fragments used to determine the parameters. The recommended restraints are printed in bold.

	PDB 1d8g (*N* = 3)	PDB 3p4j (*N* = 6)	Parkinson (*N* = 21)	CSD (*N* = 63)	QM for G	QM for CG
N1—C2	1.372 (13)	1.374 (3)	1.373 (8)	**1.372 (6)**	1.366	1.373
C2—N3	1.333 (11)	1.329 (1)	1.323 (8)	**1.327 (5)**	1.308	1.328
N3—C4	1.343 (2)	1.348 (1)	1.350 (7)	**1.352 (6)**	1.352	1.350
C4—C5	1.378 (3)	1.383 (3)	1.379 (7)	**1.379 (6)**	1.393	1.402
C5—C6	1.428 (3)	1.416 (5)	1.419 (10)	**1.418 (8)**	1.432	1.428
C6—N1	1.384 (8)	1.384 (4)	1.391 (7)	**1.392 (6)**	1.435	1.407
C5—N7	1.382 (7)	1.389 (6)	1.388 (6)	**1.388 (6)**	1.379	1.387
N7—C8	1.305 (16)	1.308 (7)	1.305 (6)	**1.308 (6)**	1.304	1.310
C8—N9	1.378 (8)	1.378 (4)	1.374 (7)	**1.375 (6)**	1.384	1.390
N9—C4	1.372 (7)	1.380 (2)	1.375 (8)	**1.374 (6)**	1.367	1.373
C6—O6	1.239 (6)	1.249 (3)	1.237 (9)	**1.238 (7)**	1.213	1.243
C2—N2	1.329 (14)	1.342 (3)	1.341 (10)	**1.338 (7)**	1.360	1.351
C6—N1—C2	125.7 (1)	124.3 (3)	125.1 (6)	**125.4 (5)**	126.56	125.83
N1—C2—N3	122.6 (6)	124.1 (3)	123.9 (6)	**123.6 (5)**	123.25	123.34
C2—N3—C4	112.6 (4)	112.0 (3)	111.9 (5)	**112.0 (4)**	112.94	112.47
N3—C4—C5	129.0 (1)	128.3 (4)	128.6 (5)	**128.6 (5)**	128.96	129.01
C4—C5—C6	117.8 (6)	118.5 (2)	118.8 (6)	**118.9 (4)**	118.70	117.83
C5—C6—N1	112.3 (5)	112.5 (3)	111.5 (5)	**111.5 (5)**	109.60	111.54
N3—C4—N9	125.29 (3)	126.5 (6)	126.0 (6)	**125.8 (7)**	125.42	125.59
C6—C5—N7	131.5 (2)	130.4 (5)	130.4 (6)	**130.3 (5)**	130.87	131.58
C5—C4—N9	105.7 (2)	105.2 (3)	105.4 (4)	**105.6 (5)**	105.63	105.40
C4—N9—C8	106.3 (6)	106.3 (2)	106.4 (4)	**106.2 (4)**	106.11	106.24
N9—C8—N7	112.9 (5)	113.3 (1)	113.1 (5)	**113.2 (4)**	113.04	113.22
C8—N7—C5	104.4 (3)	104.2 (3)	104.3 (5)	**104.2 (4)**	104.79	104.55
N7—C5—C4	110.7 (3)	111.1 (5)	110.8 (4)	**110.8 (4)**	110.43	110.59
O6—C6—N1	119.8 (11)	120.5 (2)	119.9 (6)	**120.1 (5)**	118.92	119.67
O6—C6—C5	127.9 (6)	127.0 (3)	128.6 (6)	**128.4 (6)**	131.48	128.79
N2—C2—N1	117.2 (3)	116.5 (2)	116.2 (9)	**116.5 (6)**	117.39	116.61
N2—C2—N3	120.2 (8)	119.4 (3)	119.9 (7)	**119.9 (6)**	119.37	120.06
RMSD versus 3p4j (*d*/*a*)			0.0063/0.665	0.0062/0.674	0.0214/1.602	0.0112/0.908
RMSD versus 1d8g (*d*/*a*)			0.0112/0.851	0.0111/0.803	0.0223/1.300	0.0155/0.646
RMSD versus QM for G (*d*/*a*)						0.0144/0.935

**Table 3:** Molecular geometry (Å, °) of N1-substituted uracil *N* denotes the number of CSD fragments used to determine the parameters. The recommended restraints are printed in bold.

	Parkinson (*N* = 46)	CSD (*N* = 180)	QM for U	QM for AU
N1—C2	1.381 (9)	**1.381 (9)**	1.392	1.400
C2—N3	1.373 (7)	**1.373 (8)**	1.378	1.376
N3—C4	1.380 (9)	**1.381 (8)**	1.404	1.389
C4—C5	1.431 (9)	**1.432 (8)**	1.450	1.451
C5—C6	1.337 (9)	**1.337 (8)**	1.345	1.355
C6—N1	1.375 (9)	**1.374 (8)**	1.371	1.371
C2—O2	1.219 (9)	**1.219 (8)**	1.214	1.225
C4—O4	1.232 (8)	**1.231 (8)**	1.215	1.236
C6—N1—C2	121.0 (6)	**121.1 (5)**	121.27	121.07
N1—C2—N3	114.9 (6)	**114.9 (6)**	114.26	115.27
C2—N3—C4	127.0 (6)	**127.0 (5)**	128.25	126.84
N3—C4—C5	114.6 (6)	**114.5 (6)**	112.95	114.71
C4—C5—C6	119.7 (6)	**119.7 (6)**	119.89	119.31
C5—C6—N1	122.7 (5)	**122.7 (5)**	123.38	122.80
O2—C2—N1	122.8 (7)	**122.8 (7)**	122.30	121.28
O2—C2—N3	122.2 (7)	**122.3 (6)**	123.44	123.46
O4—C4—C5	125.9 (6)	**126.0 (7)**	126.60	124.62
O4—C4—N3	119.4 (7)	**119.5 (7)**	120.45	120.67
RMSD versus QM for U (*d*/*a*)				0.0109/1.090

**Table 4:** Molecular geometry (Å, °) of N1-substituted thymine *N* denotes the number of fragments used to determine the parameters. The recommended restraints are printed in bold.

	PDB 1d8g (*N* = 1)	Parkinson (*N* = 50)	CSD (*N* = 358)	QM for T	QM for AT
N1—C2	1.387	1.376 (8)	**1.376 (8)**	1.386	1.396
C2—N3	1.367	1.373 (8)	**1.372 (7)**	1.378	1.377
N3—C4	1.383	1.382 (8)	**1.382 (8)**	1.399	1.385
C4—C5	1.445	1.445 (9)	**1.446 (8)**	1.459	1.459
C5—C6	1.348	1.339 (7)	**1.340 (7)**	1.347	1.356
C6—N1	1.385	1.378 (7)	**1.381 (7)**	1.374	1.377
C2—O2	1.223	1.220 (8)	**1.222 (8)**	1.216	1.226
C4—O4	1.218	1.228 (9)	**1.229 (8)**	1.218	1.240
CM—C5	1.505	1.496 (6)	**1.498 (6)**	1.502	1.500
C6—N1—C2	121.05	121.3 (5)	**121.2 (5)**	121.35	121.24
N1—C2—N3	114.59	114.6 (6)	**114.7 (6)**	113.87	114.77
C2—N3—C4	127.70	127.2 (6)	**127.1 (5)**	128.38	127.02
N3—C4—C5	115.01	115.2 (6)	**115.2 (5)**	114.01	115.66
C4—C5—C6	118.34	118.0 (6)	**118.1 (5)**	117.89	117.63
C5—C6—N1	123.23	123.7 (6)	**123.6 (5)**	124.50	123.68
O2—C2—N1	122.96	123.1 (8)	**123.0 (7)**	122.70	121.84
O2—C2—N3	122.45	122.3 (6)	**122.3 (6)**	123.44	123.39
O4—C4—C5	125.54	124.9 (7)	**125.0 (7)**	125.94	123.63
O4—C4—N3	119.43	119.9 (6)	**119.8 (6)**	120.05	120.72
CM—C5—C4	118.72	119.0 (6)	**118.7 (6)**	118.85	118.73
CM—C5—C6	122.92	122.9 (6)	**123.2 (6)**	123.26	123.64
RMSD versus 1d8g (*d*/*a*)		0.0073/0.346	0.0067/0.316	0.0092/0.684	0.0108/0.895
RMSD versus QM for T (*d*/*a*)					0.0104/1.033

**Table 5:** Molecular geometry (Å, °) of N1-substituted cytosine *N* denotes the number of fragments used to determine the parameters. The recommended restraints are printed in bold.

	PDB 1d8g (*N* = 3)	PDB 3p4j (*N* = 6)	Parkinson (*N* = 28)	CSD (*N* = 147)	QM for C	QM for CG
N1—C2	1.397 (6)	1.392 (3)	1.397 (10)	**1.395 (9)**	1.432	1.414
C2—N3	1.363 (10)	1.351 (3)	1.353 (8)	**1.353 (7)**	1.363	1.355
N3—C4	1.333 (14)	1.342 (2)	1.335 (7)	**1.337 (8)**	1.314	1.340
C4—C5	1.436 (13)	1.431 (4)	1.425 (8)	**1.424 (10)**	1.429	1.438
C5—C6	1.331 (14)	1.345 (5)	1.339 (8)	**1.338 (8)**	1.355	1.360
C6—N1	1.349 (14)	1.366 (3)	1.367 (6)	**1.365 (7)**	1.348	1.361
C2—O2	1.234 (3)	1.248 (3)	1.240 (9)	**1.240 (8)**	1.217	1.240
C4—N4	1.320 (6)	1.327 (2)	1.335 (9)	**1.330 (8)**	1.356	1.338
C6—N1—C2	120.8 (10)	120.9 (2)	120.3 (4)	**120.3 (5)**	121.08	120.35
N1—C2—N3	118.3 (11)	119.3 (3)	119.2 (7)	**119.1 (6)**	117.09	118.62
C2—N3—C4	121.0 (9)	120.1 (2)	119.9 (5)	**120.1 (5)**	120.90	121.28
N3—C4—C5	120.2 (8)	121.5 (2)	121.9 (4)	**121.6 (6)**	123.21	121.13
C4—C5—C6	118.1 (7)	117.4 (3)	117.4 (5)	**117.5 (5)**	116.20	117.09
C5—C6—N1	121.4 (10)	120.8 (1)	121.0 (5)	**121.2 (6)**	121.53	121.54
O2—C2—N1	119.8 (5)	119.0 (4)	118.9 (6)	**118.8 (8)**	117.87	117.88
O2—C2—N3	121.9 (7)	121.7 (4)	121.9 (7)	**122.0 (6)**	125.04	123.50
N4-C4—C5	121.2 (9)	120.2 (2)	120.2 (7)	**120.3 (7)**	119.62	120.99
N4—C4—N3	118.6 (16)	118.3 (2)	118.0 (7)	**118.1 (6)**	117.18	117.89
RMSD versus 3p4j (*d*/*a*)			0.0069/0.375	0.0062/0.381	0.0248/1.581	0.0117/0.937
RMSD versus 1d8g (*d*/*a*)			0.0211/1.325	0.0205/1.266	0.0285/2.053	0.0236/1.373
RMSD versus QM for C (*d*/*a*)						0.0166/1.135

**Table 6:** Molecular geometry (Å, °) of N1-substituted isocytosine

	*REFMAC*	CSD†	QM for iC	QM for iCiG‡
N1—C2	1.410	1.352 (3)	1.380	**1.385**
C2—N3	1.350	1.332 (2)	1.295	**1.324**
N3—C4	1.350	1.364 (2)	1.393	**1.375**
C4—C5	1.390	1.445 (3)	1.467	**1.461**
C5—C6	1.390	1.340 (3)	1.338	**1.350**
C6—N1	1.337	1.357 (3)	1.383	**1.382**
C2—N2	1.355	1.324 (3)	1.363	**1.340**
C4—O4	1.250	1.247 (2)	1.220	**1.244**
C6—N1—C2	120.0	120.23 (16)	117.05	**118.2**
N1—C2—N3	120.0	122.00 (18)	124.67	**122.2**
C2—N3—C4	120.0	119.65 (16)	120.86	**121.7**
N3—C4—C5	120.0	118.94 (15)	115.97	**117.3**
C4—C5—C6	120.0	118.40 (20)	120.10	**119.0**
C5—C6—N1	120.0	120.77 (18)	121.35	**121.7**
N2—C2—N1	120.0	118.74 (16)	116.78	**118.8**
N2—C2—N3	120.0	119.24 (18)	118.55	**119.0**
O4—C4—C5	120.0	121.69 (17)	122.29	**121.9**
O4—C4—N3	120.0	119.35 (17)	121.75	**120.8**
RMSD versus QM for iC (*d*/*a*)				0.0176/1.294

† ICYTIN01; N1-protonation.

‡ Suggested standard deviations 0.009 Å for bonds, 0.7° for angles.

**Table 7:** Molecular geometry (Å, °) of N9-substituted isoguanine

	*REFMAC*	CSD	QM for iG	QM for iCiG†
N1—C2	1.337	-	1.457	**1.419**
C2—N3	1.350	-	1.357	**1.351**
N3—C4	1.355	-	1.318	**1.334**
C4—C5	1.490	-	1.409	**1.407**
C5—C6	1.490	-	1.382	**1.401**
C6—N1	1.337	-	1.351	**1.361**
C5—N7	1.350	-	1.386	**1.388**
N7—C8	1.350	-	1.302	**1.312**
C8—N9	1.337	-	1.387	**1.390**
N9—C4	1.337	-	1.376	**1.378**
C6—N6	1.355	-	1.346	**1.337**
C2—O2	1.250	-	1.216	**1.254**
C6—N1—C2	120.0	-	126.42	**126.0**
N1—C2—N3	120.0	-	116.91	**119.6**
C2—N3—C4	120.0	-	115.56	**114.1**
N3—C4—C5	120.0	-	129.33	**129.3**
C4—C5—C6	120.0	-	116.86	**116.5**
C5—C6—N1	120.0	-	114.91	**114.5**
N3—C4—N9	132.0	-	126.59	**126.3**
C6—C5—N7	132.0	-	131.66	**132.0**
C5—C4—N9	108.0	-	104.08	**104.4**
C4—N9—C8	108.0	-	106.76	**106.7**
N9—C8—N7	108.0	-	113.64	**113.4**
C8—N7—C5	108.0	-	104.05	**104.1**
N7—C5—C4	108.0	-	111.48	**111.4**
N6—C6—N1	120.0	-	121.19	**119.8**
N6—C6—C5	120.0	-	123.91	**125.7**
O2—C2—N1	120.0	-	115.85	**116.6**
O2—C2—N3	120.0	-	127.24	**123.8**
RMSD versus QM for iG (*d*/*a*)				0.0179/1.272

† Suggested standard deviations 0.008 Å for bonds, 0.6° for angles.
