# Supplementary material for: Accurate geometrical restraints for Watson–Crick base pairs
Source: Acta Crystallogr B Struct Sci Cryst Eng Mater. 2019 Mar 27;75(Pt 2):235–45. doi: 10.1107/S2052520619002002 (PMC6457083; doi:10.1107/S2052520619002002)
Supplement: Supplementary file 1 [file b-75-00235-sup1.pdf]

Acta Cryst B
STRUCTURAL SCIENCE
CRYSTAL ENGINEERING
MATERIALS

Volume 75 (2019)

Supporting information for article:

Accurate geometrical restraints for WC base pairs

Miroslaw Gilski, Jianbo Zhao, Marcin Kowiel, Dariusz Brzezinski, Douglas H. Turner and Mariusz Jaskolski

Table S1 Rmsd comparisons of different QM methods.

	rmsd D3(BJ) vs M06		rmsd D3(BJ) vs D3	
	Distances (Å)	Angles (°)	Distances (Å)	Angles (°)
A	0.0050	0.259	0.0010	0.100
A in AT	0.0059	0.268	0.0011	0.111
A in AU	0.0058	0.265	0.0010	0.120
G	0.0056	0.310	0.0013	0.091
G in GC	0.0063	0.288	0.0014	0.067
iG	0.0050	0.254	0.0023	0.089
iG in iGiC	0.0056	0.307	0.0011	0.113
U	0.0044	0.163	0.0014	0.064
U in AU	0.0051	0.148	0.0014	0.066
T	0.0043	0.269	0.0012	0.074
T in AT	0.0058	0.254	0.0012	0.109
C	0.0045	0.209	0.0009	0.069
C in CG	0.0051	0.212	0.0010	0.048
iC	0.0040	0.209	0.0014	0.124
iC in iGiC	0.0046	0.155	0.0015	0.096

Table S2 CSD refcodes of structures used to determine the geometry of the indicated nucleobases.

**adenine**
ACADOS ACUHUB ADENOS01 ADENOS10 ADIJEC ADOSBU01 ADPCPY ADPROP ADYPUR10 AJAFIA AMDOAD AMOADA AMOADB AMOADC ARADEN10 ASOZIS ATEXOM AWAZAA AWAZEE BAPTOE BEDLIF BETWUS BIFYOE BOYDUO BUVPOX CAFKEA CAQLUC CAQMAJ CATGIP CATGOV CATGUB CELLIP CEQYON CEQZEE CEZBIS CEZMEZ CHXADI10 CIDGUR COCZID CORDCP01 CUBRUM DAHJUU DATZOP DATZUV DHOADS01 DHOADS10 DOHGEM DORJOJ DOXADM02 DPHEAD20 DUXSAR EADPBA EGONET EWOCUP FABFUJ01 FADSAF FEXSIL FEYBER FIBGEC FIKCAE FIKCEI FIKHAI FIKHAI01 FISKOI FOYLUA FUBZEJ FUWTOH GAHHIG GAHHIG01 GIQRIH GODPIY HACNUV HACPAD HAHKEG HAXWOV HEMYIH HIFKEM HIQNIE HOZBED HOZTAT HUGXUD HUVGIP HUVGOV HUYCEK IKOJEX IKOJOH IPADOS02 ITOLOU02 IVAMUO IZECAS JACTUD JACVAL JADMUX JAHLAF JESVOT KECYUN KECZAU KEMYEG KEMYEG01 KEPLIA KEPLOG KEWGAV KUBGUJ KURGEK KUYWEH LAPXEH LOQROY MARBEM01 MEADEN01 MEADEN02 MEADEN04 MEADEN05 MEDLEN MICFIP MICFOV MICGAI MOPJEG MTHMAD12 MTHMAD13 NAAMPH10 NAGLAI NAKPUN NAKQAU NAKZUU OJEJES OJUTET OPADNA OPADNA01 OPADNB ORELAA ORELEE OVAZAN PAFBUV PAKWON PEXTAN PIVSUI QACLUE QACMAL QAWTUE QETWIV QICLAP QIWGAE RITNAK RUBVEQ RUHRES SAYQUE SICHES SITMIR TELBOD THOPAD10 TIKQIP TIKQUB TOYTAE TOYTIM TUGJAG TUHCEF TUNMAQ UNALEB URALOQ VAVTOB VAVZUO VAZRUL VOMFOS VORQOK WAJZEM WEZZUY WIBXEK WIPCIH WIPWEX WOBXAM WULXOQ WUSGOH WUWCUO XAKTIM XASFED XECLEX XIJPUD XOBRIQ XUDQAP XUXQIS YABGAJ ZEVTID EWOCUP01 ACEQEH ADENOS13 REFXAE FEXNEE NAAMPH11 LEYBID
**guanine**
ANITUN BUDWAY10 CAQKOV CAQLIQ CEHTAK10 CELLOV EMUHAX EMUHEB FOZNIT GEBRIO GIPBIQ GUANSH10 GUOPNA10 GUOPNA11 HOPBOD JAFHIH KUTDOU MECWIC MECWOI NEPLID OXIJEM QUQWOO RECZAA RIGDES SCGMPT10 SCGMPT11 SDGUNP TAMXEK TARLAC VIDYOY VUWREK WULXUW XIZGOC YACGUE YACGUE01 YIHJOP YIHJUV YIHKAC YURTIP ZEVPEX ZEVPIB ZEVPOH ZUZXAT UGIVAI01 BEWKUM BEWKUM01 KETNEF KETNEF01 KETNEF02 WIFDUN
**uracil**
AFOBAZ AURCPB AXEFOA BENXEX BEPFEI BEURID10 BINRIB BIYRIK BOFWIC BOPVOS BUDVUR10 BUFYIK CAWCAE CDURID CEDBIW CEKLUZ CIHHIK01 CIRYUX COMBOV CUDYOR CUKQAB CUZWIE DAURID01 DAYPID EHOQOJ EHOREA EHUFEU EHUFIY EJAFOM ERAJUC EVIPOO FECFAU FICJEI FIPKAQ FIPKEU FISKIC FIXMIK FIYTAJ FIYTIR FUNTEN GATHOY GIDZIC10 GIYWOB GIYXIW GULVEO HALRES HEXWAJ HIKNIY HURLIQ IGORIF IJIWIH JEVPAC JEYKOO JEYKUU JIKBUA JIQREG JIQRIK JUCNOK JUCPAY KATYOT KIGMEU KIGMOE KIHCIO KIXFON KOFGUH KOFGUH10 KUKSEQ LAVXOW LOWJIQ LUWDOY LYFURA MAQVAB MAWPUW METURA01 METURA02 METURA03 METURA04 METURA05 MOPJIK MUTWED MXEURD NIFNOF OJOVOZ OXIGAE PABKOS PARZIU PEHZAF PEQDEU PETQIP PIRZAS PUDVAN QAHZAB QAJJES QAKBOV QAVYES QETWER QEXQUG QIXYOL QUBVUE

	RAQDUI ROVQEA RUHDED SAHDIP SAPSIL SIJGID SURIDP SUROMM THFURC10 THPYUR TIMKEH TULCIO TUWMOP ULENOQ URARAF01 URARAF10 URIDMP10 VAHDEQ VAHDOA VANJEZ VAVZIC VEVHUZ VEVHUZ01 VOMFIM VOMGAF VOYVAG VOYVEK VUKGOX WAXLEM WEVJOX WIBWIN WIRZIG WIVYUV WIVZAC WOBKOO WORVUW XAKNON XECLIB YEDHEU YERDAA YERDOO YERDUU YIBDOC YISCIO YOFMUC YOGYOI YOTWOV YOTXIQ ZAMTEM ZAPXIX ZZZAPA10 CEWJAS HENHOA XEVBIM LIGXIL
**thymine**	
	ABEPIH ABEPIH01 ABIRIP ABUKOX ACOJOT AKUNAW AKUZEM ALALAB AMERUF AQEJUC ASABAX ASABEB ASACIG ASACOM AWAZII BICRUA BICRUA01 BINQUM BINRAT BINREX BISLAQ BOLVED BOPQOO CACPAY CEVWOR CEWXAE CEZFOC CIMHIR CIXZOZ CLQUNB10 COJZOS COJZUY COKBAH COKBEL COKBIP COWLIL CUQBOG DAYVOO DEJSAM DELCUU DEZNON DISLUN DOXZIZ DUKSOR DUNSAH DUXXEZ ECIJOR ECIKAE EHABAS EHABEW EHABIA EHOROK EXUPES FEFMEK FIRPOL FIXGAU FIXGAU01 FIXGAU02 FIXGAU03 FIXGAU04 FIXGAU05 FIZMAD GAKSEQ01 GEBTEM GEHVOF GEXXIQ GEXXIQ01 GINKOE GIYWIV GIYXES GUYVAX HANPOB HAXWOV HIKCAH HIXKIK HUVGEL IFIGAG IHOHEU IWUKOB IYATIL IZUXAC JACVIT JEYLUV JUBQUU KASVEF KATYIN KATYIN01 KAZBIZ KAZBOF KECYUN KEDPOB KEXFUO KEXKIH KEZRUC KICJEL KIFDAF KINHEU KINHIY KITSOV KOGBIR KONNOQ KOTCOL KUNNEM KUPVOG KUTDUA KUTHOW KUZXEI LEDRAN LEDRER LEPVOR LESJUP LETJEZ LOWHEK LOWHIO LUWDAK LUWDIS LUWDOY MABWAP MAWBIV METHYM01 METHYM03 METHYM04 MEURID02 MEURID03 MIBYUS MISQOU MOGMAW MTHMAD12 MTHMAD13 MUTWUT NAGLEM NIJKEX NIJKIB NIXHEH NOFSIM NOMKUV NOMLAC OBUCIA OBUCOG OGETAV OKETUT OLATAW ONIYUH OXIGEI PAFSUN PAFTAU PAGLEQ PAGLOA PALWUZ PEYQUG PEYRAN PIZNIV POPCEE PUKQOB PUMCOR QAJYEI QITKOU QOKJUV QOVFAK QUMNUH RALWOR RAZKOT REGHER REZTEV REZTIZ REZTOF RIGTEG RIGTIK RULHEM SARKUU SENXUG SEPKOO SODDEU SOSBEI SOSBIL SULRIA TEFFOB TEKTOS TEYDAC05 TEYDAC06 TEYDAC07 THYDIN01 THYDIN02 THYDIN03 THYDIN05 THYMDN01 TICSAZ TIKQOV TIKRAI TOLXUO TPATAA TRFUTM TUSZIR TYMCXA UPAQOT UTAXEU UTEWEX UTEWIB VABVID VAHDIU VAHNEZ VASZOF VAVZOI VEVBUT VEVBUT01 VEVROE VIGGEX VIGGEX01 VIKFEA VIYSUS VUWCUM WECGES WELMEF WEYRUP WIPCED WIPCON WIXHUG WIXJAO WOMFUA WOMGAH WOMGIP WOMHEM WUQJIB WUQZAJ XACMUM XEBJAQ XEGMOL XEWQUL XEZCAH XOSGOC XOSGUI XOSHAP XOSHET XOSLUN YACTEC YACTEC01 YACTEC02 YAFQEC YAFQEC01 YAPGON YASJIM YAVKUB YEGSEI YEMZUN YEWCEJ YOGYEY YOGYIC YUYYIA ZABSAW ZASQUF ZASRAM ZEXWII ZUGRIC ZUNQEG ZUVTIV CELDAB JELBEK JELBIO REJHOG REJJAU CEWCAL HENHUG
**cytosine**	
	ACYTID AMAJON AMAKAA BAGXIQ01 BARZOJ BARZOJ01 BISMAR BIVVIL BOFWOI BOMTAY BOSGOF BOTSIM BOTSIM10 BOXGIE BUDWAY10 CIMJEN CITMOH01 COQNAX CUPYUH CYTCYP20 CYTIDI01 CYTIDI02 CYTIDI10 CYTIDI11 CYTIDI12 DATZIJ DECYUF DXCYTD DXCYTD01 FIKHOW FIYTEN FOCFOT FOVXET GAHHOM GAHHOM01 GIGMOY GODJEQ GOKFES GOUGER10 GUTPUI HIMXIL HIXKOQ HUYCAG ITOCUQ JIKHOA JUHLAZ KAYWEN

KECCUT KECRUI KEWGEZ KIDLUF KIDMAM KIGMIY KOSNOV LALKOZ LAVZEO MARAFC
MAZFUO MAZGAV MCYTIM10 MECTSI METCYT01 NAGLIQ NUJDAX NUWKEX PEHYEI PEHYOS
POGGAT PUCPAF QECCIK QOMQEO QUWXOW RIQNAH RIVCAA RIVCEE RUCPUB RUKGUZ
RUKHAG01 SEGMAS SETWIY SIKBOE SIVZUS SOMVUL UDOVIT UPEPUB UPEQEM UYEZAB
VISWAX VUVNIL WAXLUC WIPWIB WOMHEM XANBAQ XANBEU XAXPIW XEGXUC XOFZAV
XOMKOB XOMKUH XUHZEF XUHZIJ XYFCYT10 YUKLIZ ZEKGII TEVYIF TEVYOL

Table S3 Molecular geometry (Å, °) of methylated A/G and adenosine/guanosine.

	methyl-A	ribo-A	methyl-G	ribo-G
N1-C2	1.338	1.341	1.366	1.369
C2-N3	1.331	1.334	1.308	1.308
N3-C4	1.334	1.336	1.352	1.359
C4-C5	1.394	1.396	1.393	1.393
C5-C6	1.405	1.408	1.432	1.438
C6-N1	1.340	1.343	1.435	1.443
C5-N7	1.380	1.382	1.379	1.378
N7-C8	1.309	1.310	1.304	1.306
C8-N9	1.377	1.386	1.384	1.390
N9-C4	1.375	1.380	1.367	1.373
C6-N6	1.349	1.356	-	-
C6-O6	-	-	1.213	1.211
C2-N2	-	-	1.360	1.376
C6-N1-C2	118.83	118.47	126.56	126.57
N1-C2-N3	128.36	128.61	123.25	123.36
C2-N3-C4	111.59	111.47	112.94	112.90
N3-C4-C5	126.56	126.72	128.96	128.98
C4-C5-C6	116.11	115.91	118.70	118.83
C5-C6-N1	118.55	118.82	109.60	109.35
N3-C4-N9	128.18	128.28	125.42	125.45
C6-C5-N7	132.99	132.86	130.87	130.45
C5-C4-N9	105.26	105.01	105.63	105.56
C4-N9-C8	106.00	106.09	106.11	105.88
N9-C8-N7	113.70	113.41	113.04	113.01
C8-N7-C5	104.14	104.26	104.79	104.81
N7-C5-C4	110.91	111.23	110.43	110.72
N6-C6-N1	119.09	118.94	-	-
N6-C6-C5	122.36	122.24	-	-
O6-C6-N1	-	-	118.92	119.12
O6-C6-C5	-	-	131.48	131.52
N2-C2-N1	-	-	117.39	117.06
N2-C2-N3	-	-	119.37	119.53

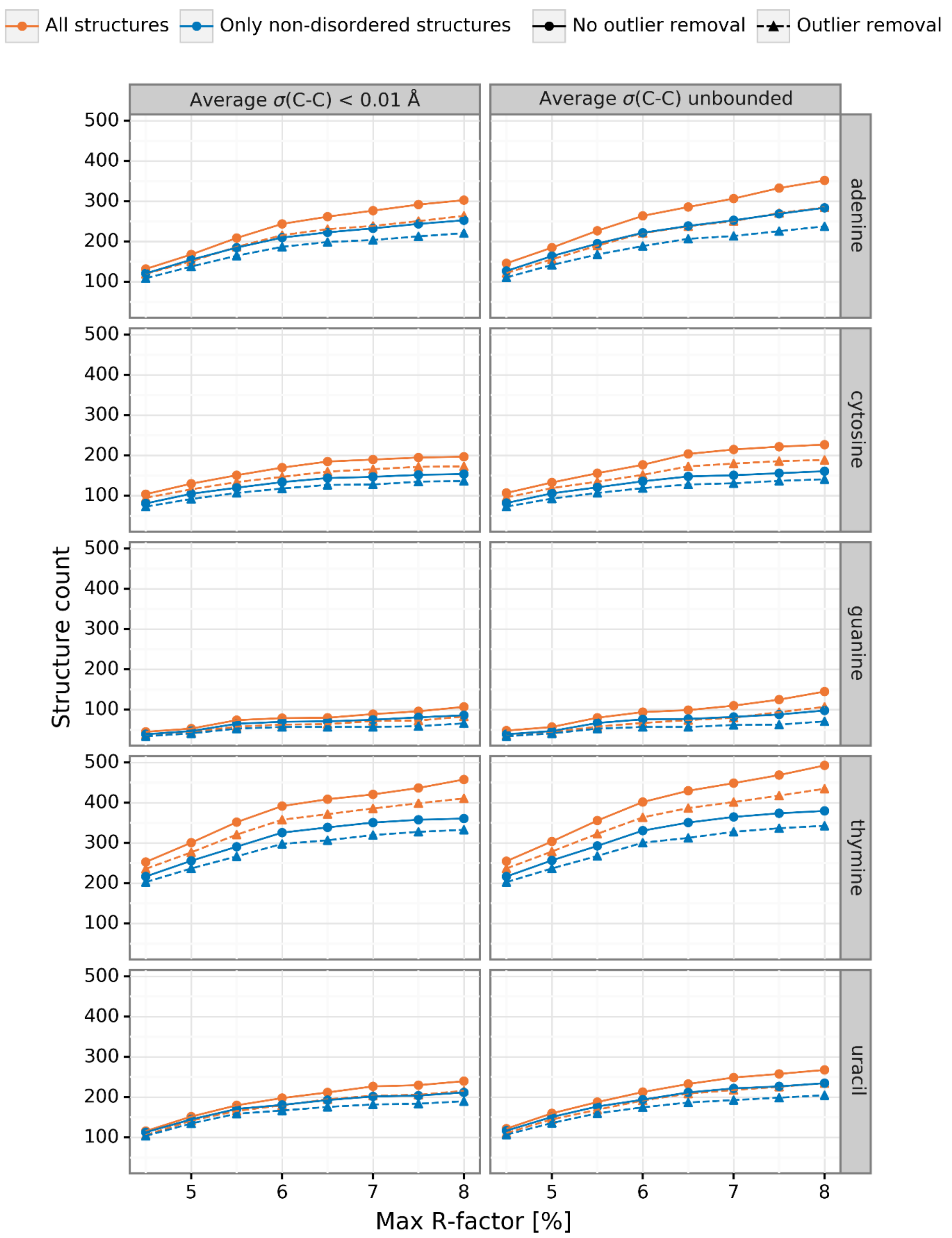

Figure S1 Number of nucleobase substructures in the CSD retrieved using various sampling criteria: maximum *R*-factor threshold (x-axis), maximum σ(C-C) (columns), all/non-disordered structures (blue/orange), all structures/outlier removal (solid/dash line).

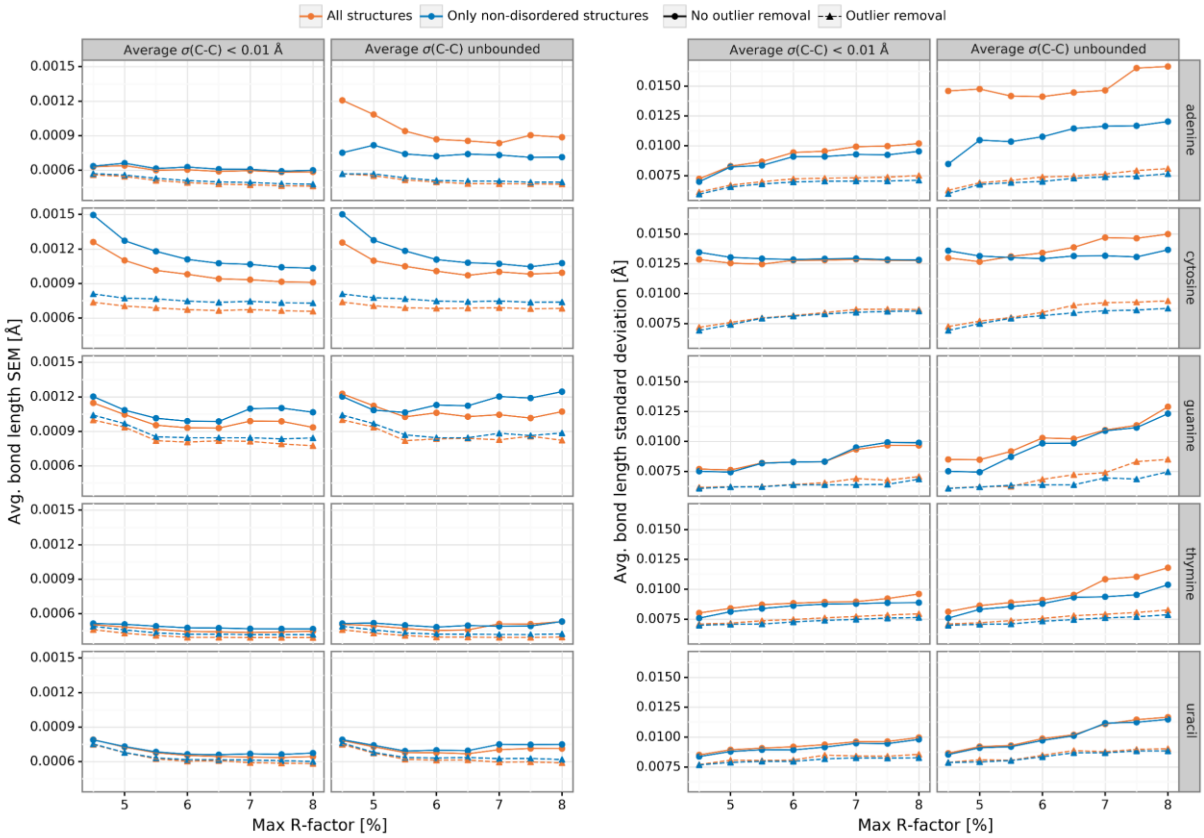

Figure S2 Analysis of average bond length (Å) standard error of the mean (SEM) (left) and sample standard deviation (right) for varying CSD sampling criteria: maximum *R*-factor (x-axis), maximum σ(C-C) (columns), all/non-disordered structures (blue/orange), all structures/outlier removal (solid/dash line).

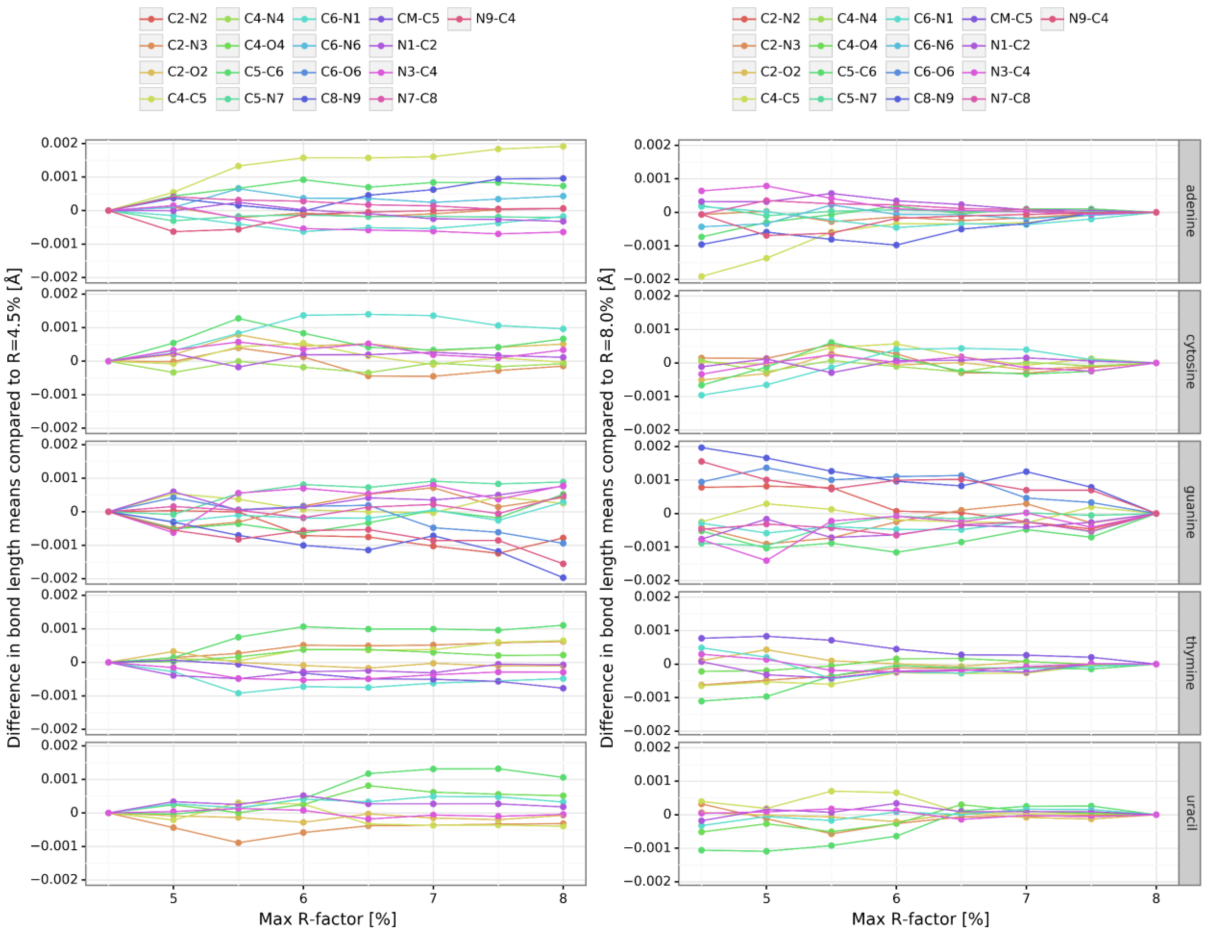

Figure S3 Deviations of the mean values of bond length (Å, left) and bond angle (°, right) calculated for CSD samples with different *R* thresholds, with reference to the set with *R* ≤ 4.5% (lowest *R*-factor).

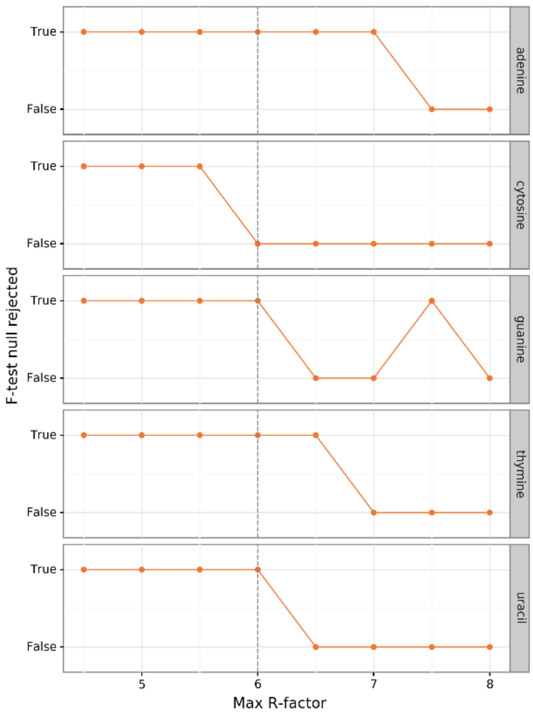

Figure S4 Results of *F*-test comparing the variance of CSD structures having *R*-factor ≤ 8% with samples having lower max *R*-factor, at significance level 0.05.

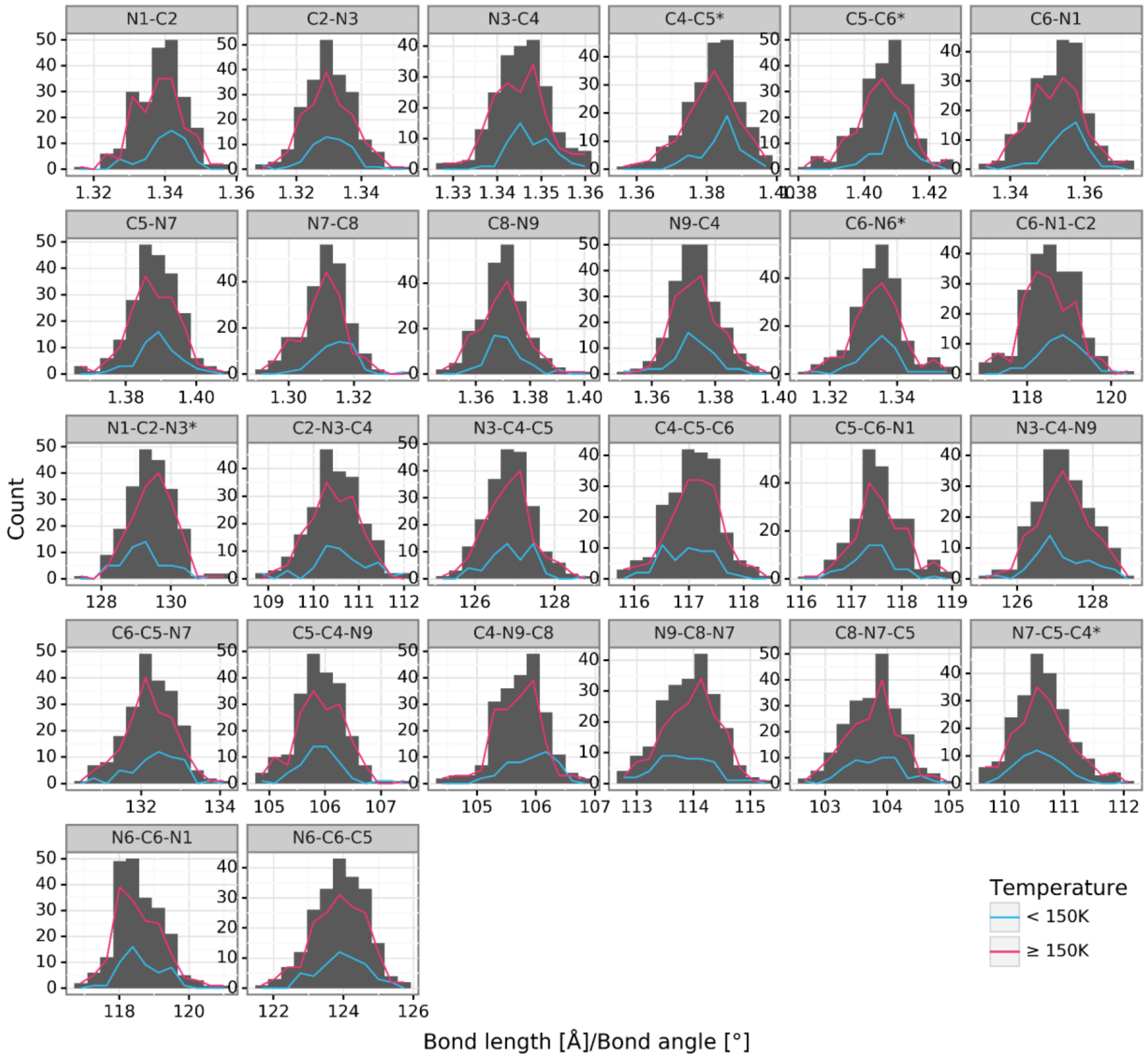

Figure S5 Frequency distributions of bond lengths (Å) and angles (°) for adenine. Bond lengths and angles with non-normal distribution according to the Shapiro-Wilk test at significance level 0.05 are marked with an asterisk.

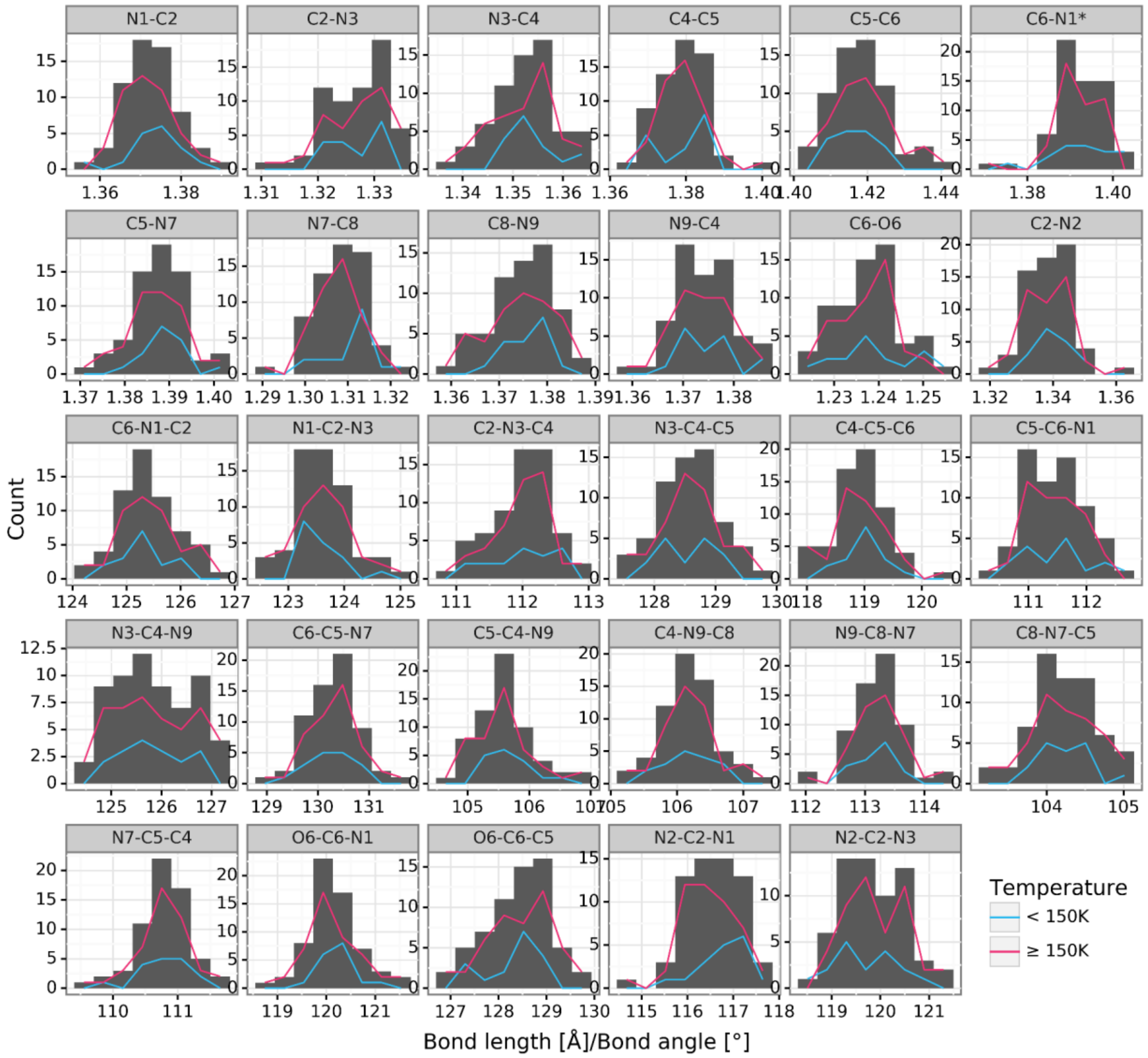

Figure S6 Frequency distributions of bond lengths (Å) and angles (°) for guanine. Bond lengths and angles with non-normal distribution according to the Shapiro-Wilk test at significance level 0.05 are marked with an asterisk.

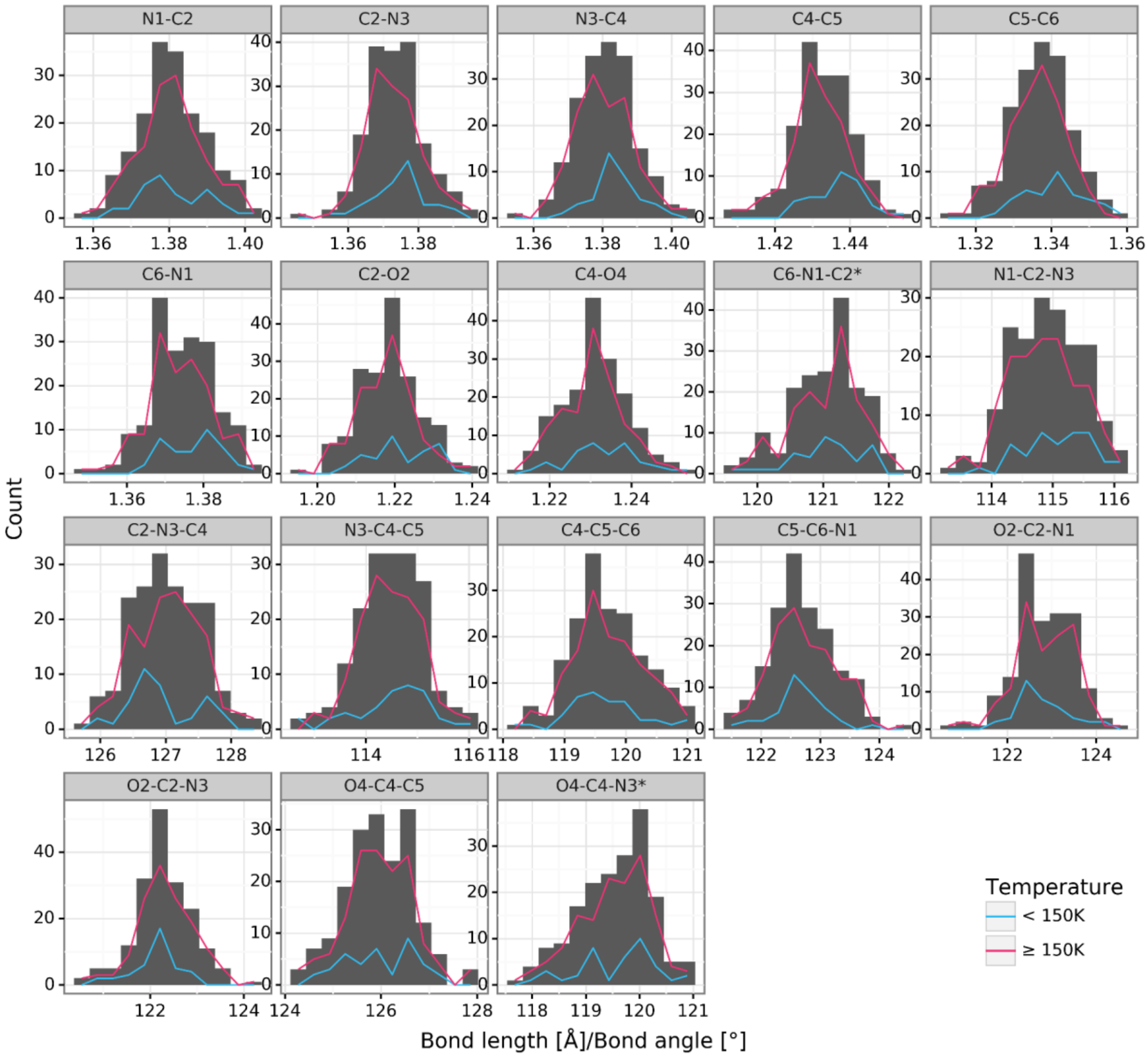

Figure S7 Frequency distributions of bond lengths (Å) and angles (°) for uracil. Bond lengths and angles with non-normal distribution according to the Shapiro-Wilk test at significance level 0.05 are marked with an asterisk.

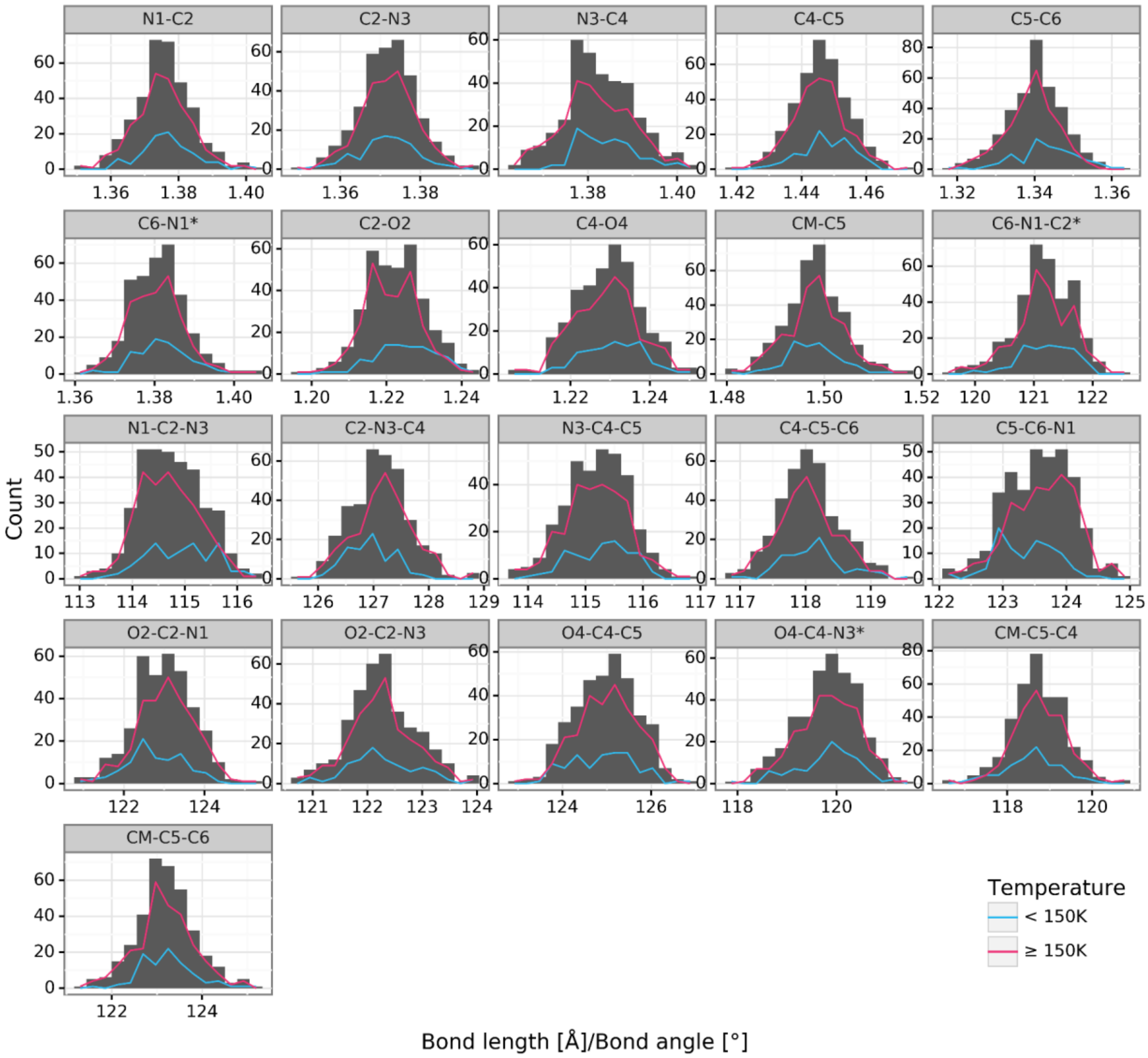

Figure S8 Frequency distributions of bond lengths (Å) and angles (°) for thymine. Bond lengths and angles with non-normal distribution according to the Shapiro-Wilk test at significance level 0.05 are marked with an asterisk.

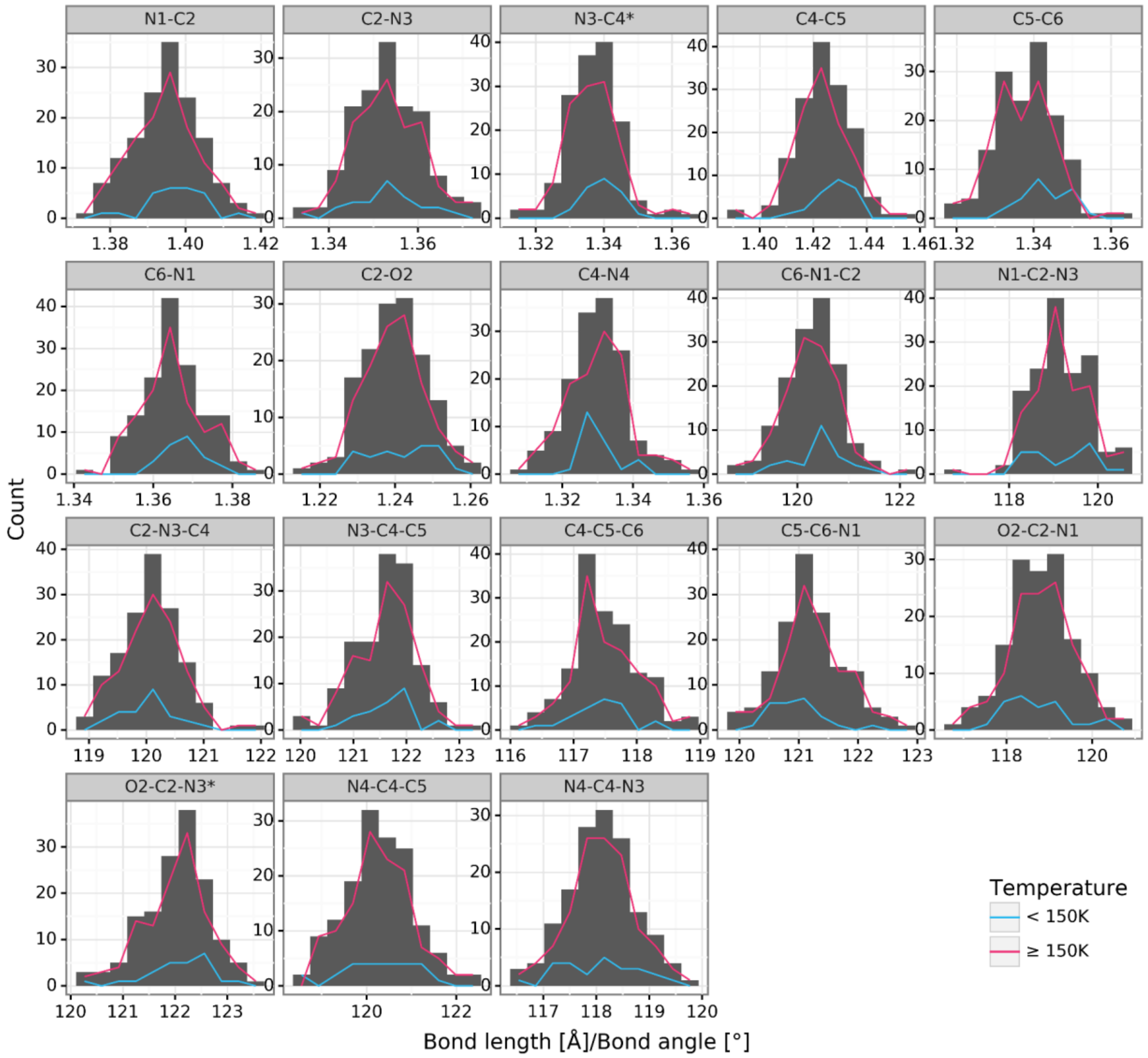

Figure S9 Frequency distributions of bond lengths (Å) and angles (°) for cytosine. Bond lengths and angles with non-normal distribution according to the Shapiro-Wilk test at significance level 0.05 are marked with an asterisk.

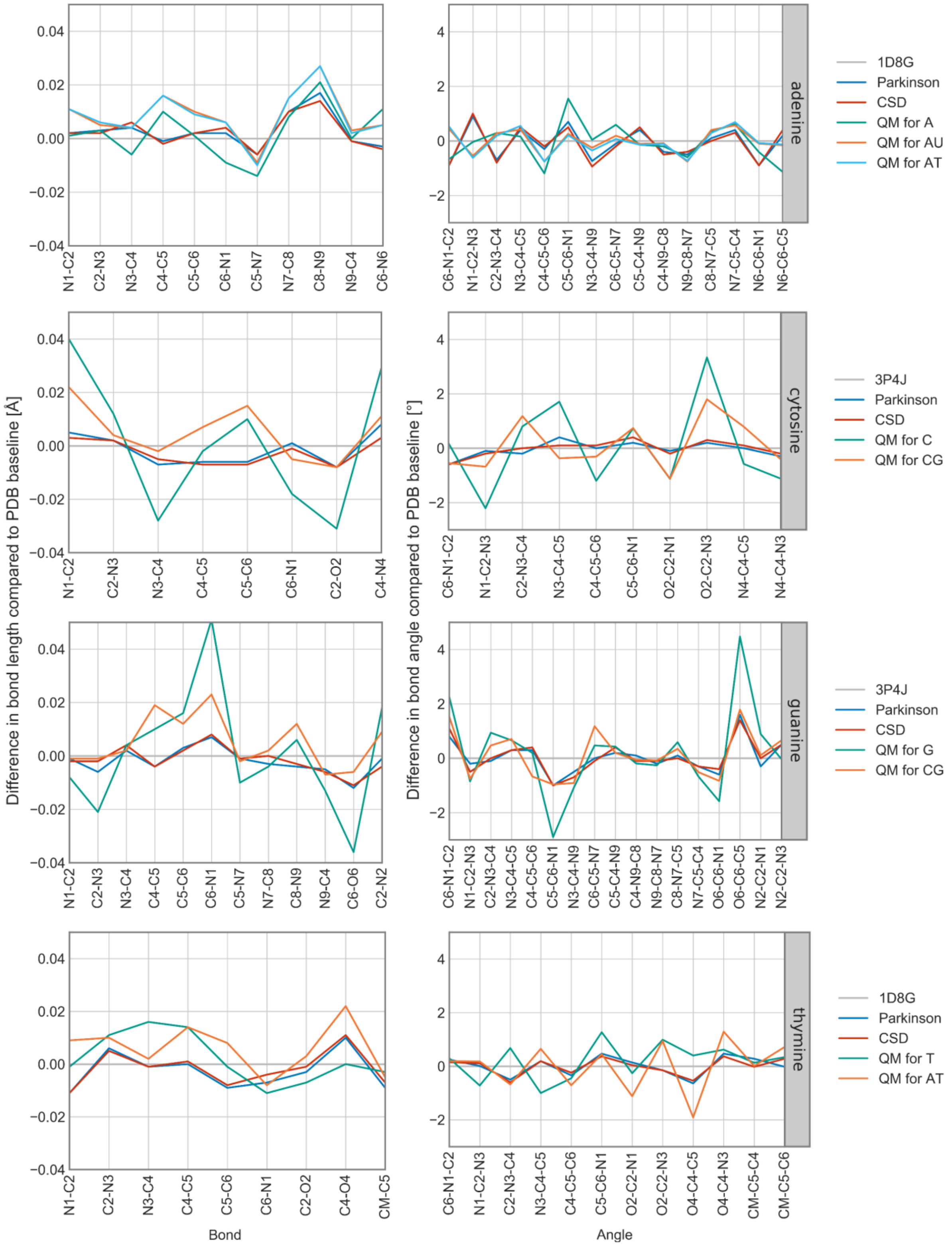

Figure S10 Parallel coordinates plot comparing Parkinson, CSD, and QM restraint targets against PDB baselines (3p4j /1d8g). Each set of restraint targets (Parkinson, CSD, QM) is represented as a set of connected line segments. Each vertical line represents one bond or angle. The y axis shows the differences in bond lengths (Å) and angles (°).
